# Supplementary material for: Multiple origins and phenotypic implications of an extended human pseudoautosomal region shown by analysis of the UK Biobank
Source: Am J Hum Genet. 2025 Feb 20;112(4):927–39. doi: 10.1016/j.ajhg.2025.01.026 (PMC12081224; doi:10.1016/j.ajhg.2025.01.026)
Supplement: Document S1. Figures S1–S7 [file mmc1.pdf]

The American Journal of Human Genetics, Volume *112*

Supplemental information

Multiple origins and phenotypic implications of an extended human pseudoautosomal region shown by analysis of the UK Biobank

Nitikorn Poriswanish, James Eales, Xiaoguang Xu, David Scannali, Rita Neumann, Jon H. Wetton, Maciej Tomaszewski, Mark A. Jobling, and Celia A. May

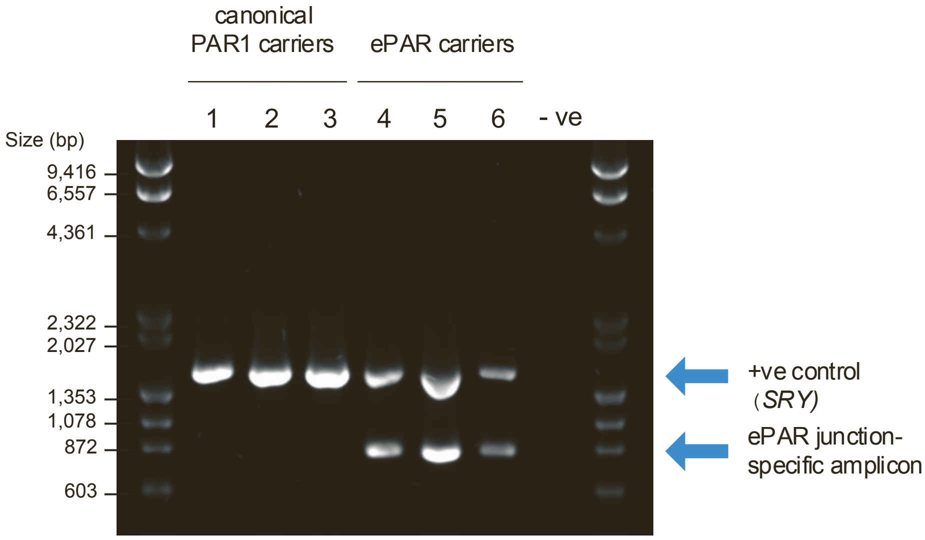

Figure S1: PCR assay for the detection of ePAR-bearing Y chromosomes.

Ethidium-stained agarose gel showing PCR products generated in three normal (canonical PAR1) males and three ePAR-carrying males. The smaller PCR product derives from the ePAR junction sequence, and the larger from the Y-specific *SRY* gene, acting as control for successful PCR.

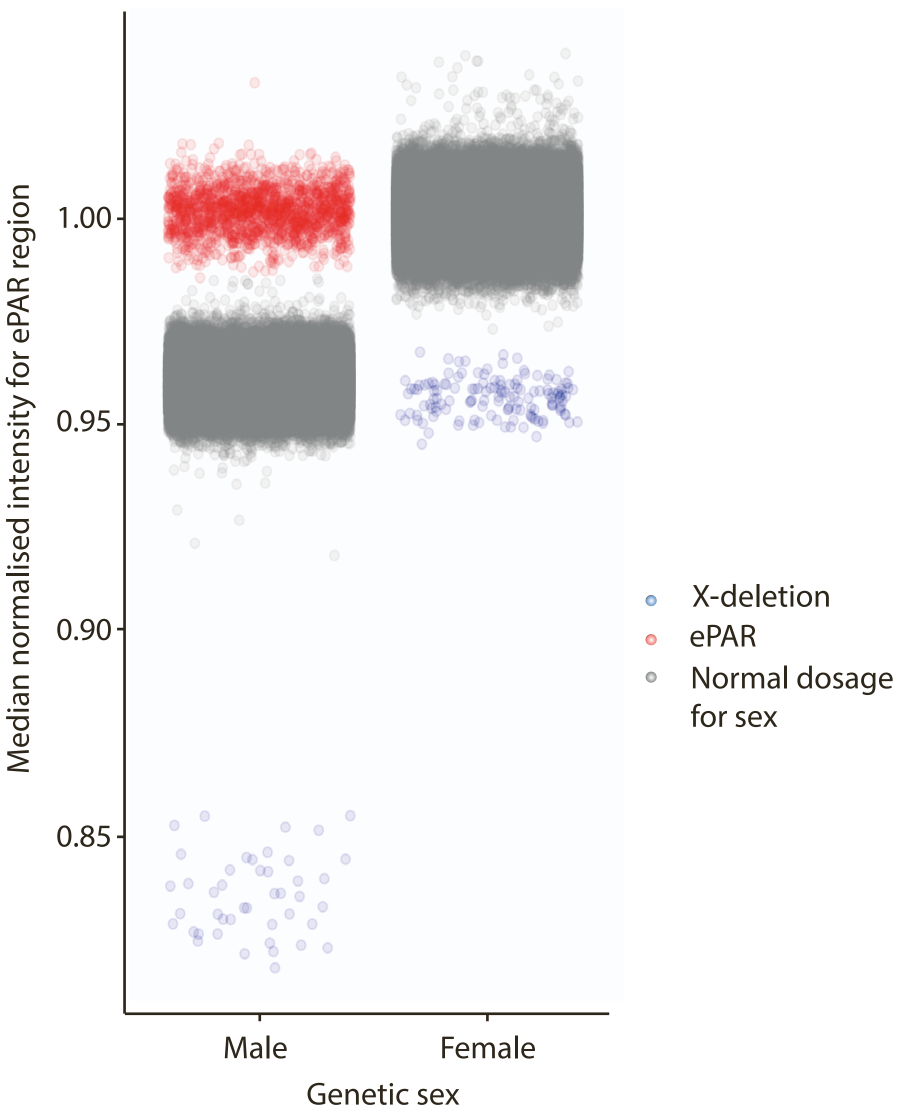

Figure S2: Median normalised intensity data for ePAR-informative SNPs amongst UK Biobank 46,XY males and 46,XX females.

The cloud of blue points in females signifies likely heterozygous carriers of the X-deletion (n=137). No female homozygotes are evident. Compare to Figure 2, in which males are subdivided by Y-hg.

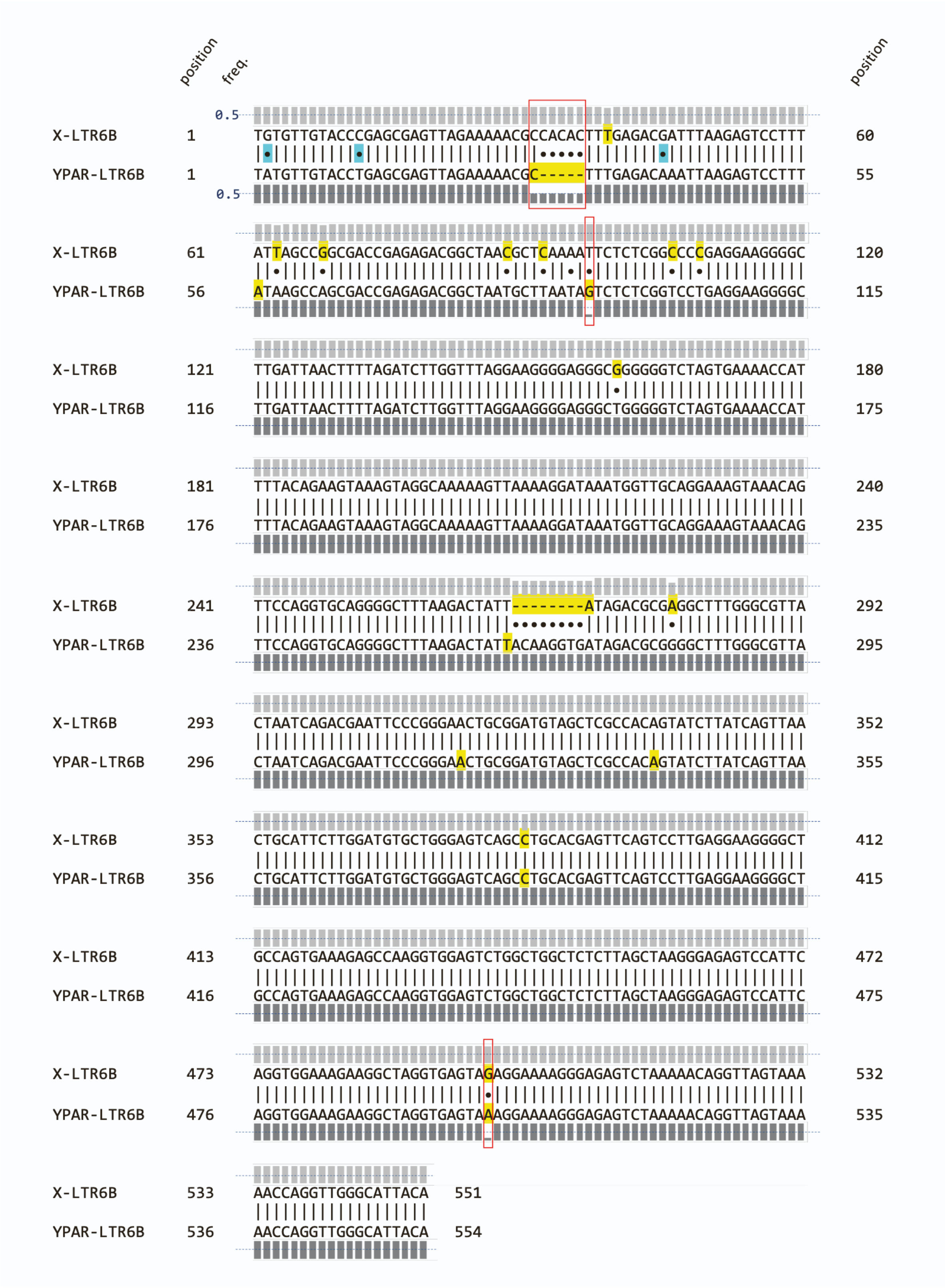

Figure S3: Alignment of the LTR6B elements from the X chromosome and Y-PAR that are implicated in the generation of the ePAR.

Alignments are shown between the hg38 genome references chrX:2,890,506-2,891,056 and chrY:2,776,108-2,776,661 with the associated histograms indicating the frequency of each base. Mismatches highlighted in cyan correspond to fixed differences between the two elements and nucleotides highlighted in yellow correspond to sites of known variation observed from the 1000 Genomes Project. The red boxes highlight instances where a reference sequence is most likely to lead to misinterpretation of NAHR-mediated junctions within ePAR candidates and the reciprocal deletion events.

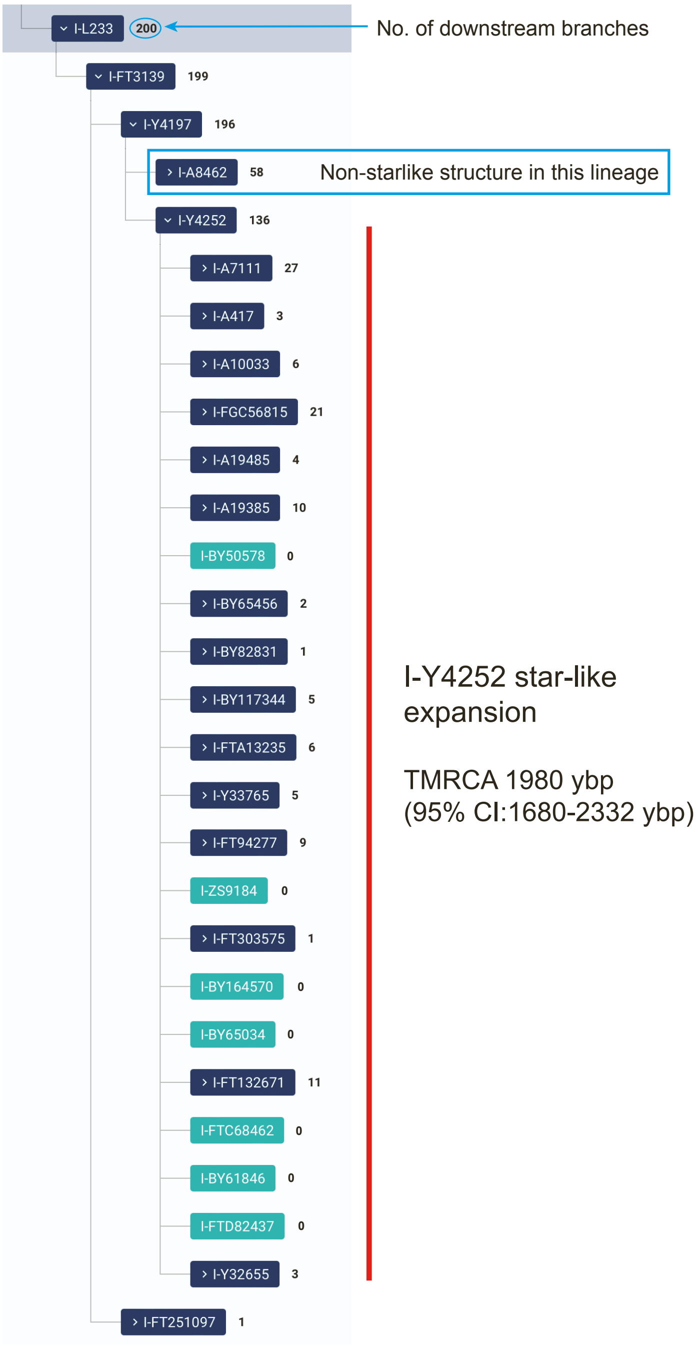

Figure S4: High-resolution SNP phylogeny for the major ePAR-bearing lineage I2-L233.

Part of the FamilyTreeDNA Y Haplotree showing the Y-Hg I2-L233 phylogeny based on ‘Big Y’ direct-to-consumer testing, which sequences 16-22.5 Mb of Y-chromosomal DNA for SNP discovery. Sublineages are named by their terminal SNPs. A striking star-like expansion, containing at least 26 distinct sub-lineages (the 22 shown, plus four as yet unnamed sub-lineages) descends from the I-Y4252 node in the Haplotree. Dates for the TMRCA of this expansion (estimated by FamilyTreeDNA using a relaxed clock model) match closely those estimated independently in this study for Y-Hg I2a_L233 from PPY23 Y-STR haplotypes. The precise position of the ePAR occurrence in the phylogeny is unclear, but we have observed ePAR carriers among those derived for SNP Y4252 and A8462. This lineage shares a common ancestor, Y4197, with a TMRCA of 150 BCE, which accounts for 98% of typed I-L233 individuals (data not shown). The contributing DNA donors have NW European / British Isles ancestry where this is stated.

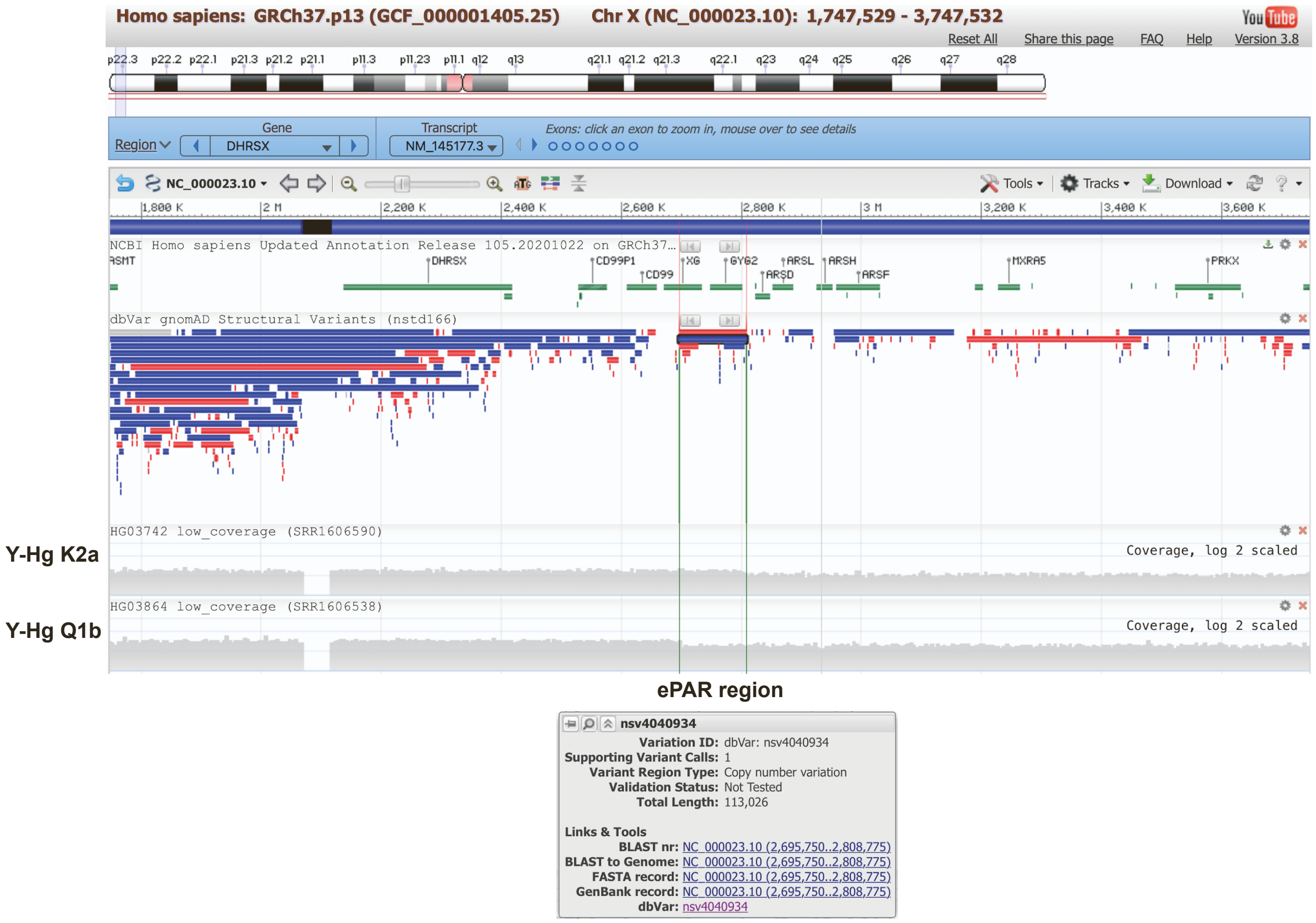

Figure S5: ePAR in a Y-Hg K2a 1000 Genomes Project male detected via sequence coverage

A screengrab of the 1000 Genomes Project browser (ncbi.nlm.nih.gov/variation/tools/1000genomes/ - now retired, but accessed 25/06/2021) centred on the interval corresponding to ePAR. HG03742 (Y-Hg K2a) shows elevated sequence coverage in this interval compared to a control (HG03864; Y-Hg Q1b). Haplogroup information is from Poznik et al. (2016).

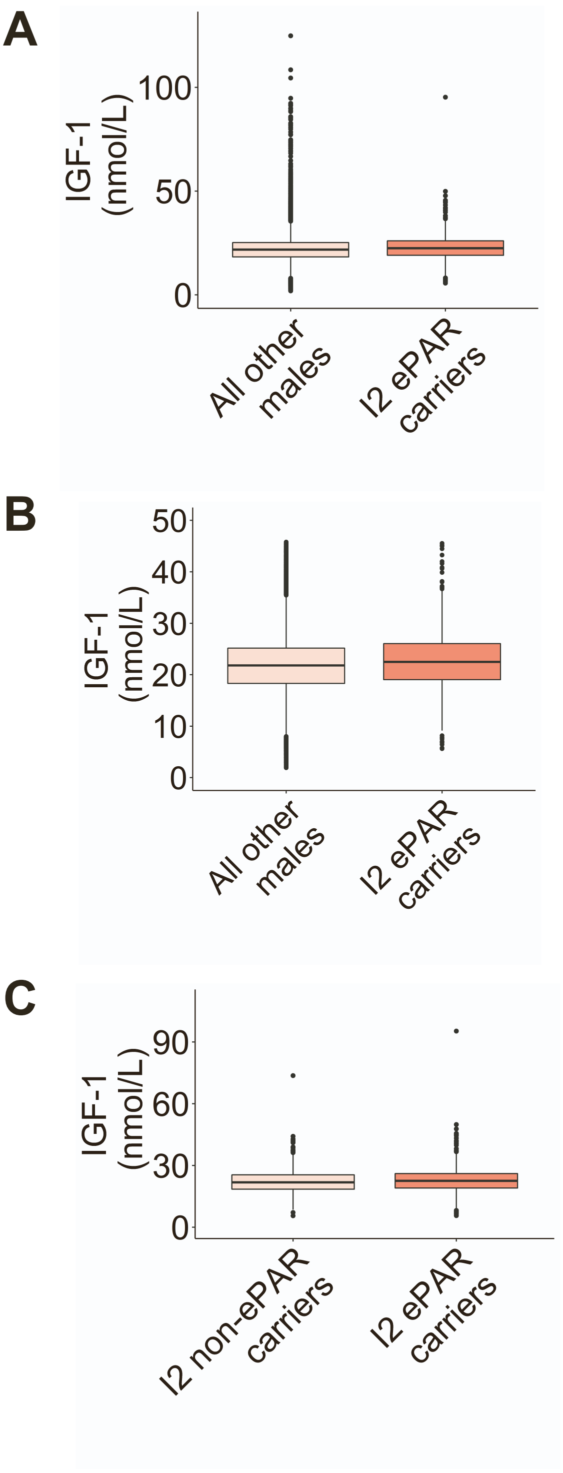

Figure S6: Levels of IGF-1 in I2 ePAR carriers compared to non-ePAR carriers.

A: Circulating IGF-1 levels in I2 ePAR carriers compared with all other non-ePAR men in the UKBiobank cohort. **B**: As (A), but excluding outliers. **C**: Persistence of the effect when comparisons are restricted among White British men of Y-Hg I2. Statistics accompanying these comparisons are given in Table S8.

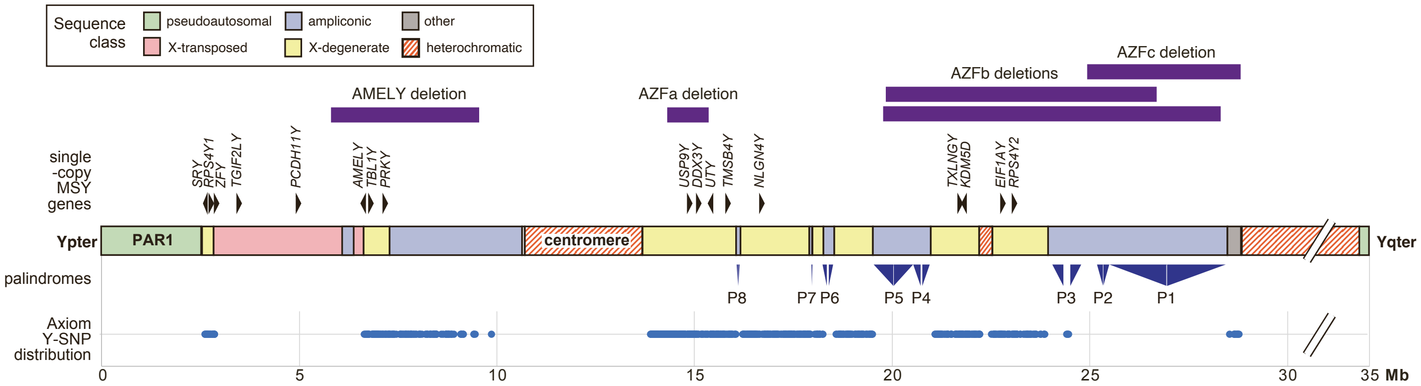

Figure S7: Distribution on MSY of Y-SNPs on the UK Biobank Axiom array.

Positions of the 813 Y-SNPs are shown relative to a schematic MSY structure based on that of Skaletsky et al. (2003). Also shown are the positions of single-copy genes, and known large deletions. The AZFc deletion will not be detectable based on SNP array data due to a lack of SNPs in the deleted region.
