# Supplementary material for: Multiple origins and phenotypic implications of an extended human pseudoautosomal region shown by analysis of the UK Biobank
Source: Am J Hum Genet. 2025 Feb 20;112(4):927–39. doi: 10.1016/j.ajhg.2025.01.026 (PMC12081224; doi:10.1016/j.ajhg.2025.01.026)
Supplement: Document S2. Article plus supplemental information [file mmc4.pdf]

Multiple origins and phenotypic implications of an extended human pseudoautosomal region shown by analysis of the UK Biobank

Authors

Nitikorn Poriswanish, James Eales, Xiaoguang Xu, ..., Maciej Tomaszewski, Mark A. Jobling, Celia A. May

Correspondence

maj4@le.ac.uk (M.A.J.), cam5@le.ac.uk (C.A.M.)

The 2.7-Mb pseudoautosomal region (PAR1) is vital for meiotic sex chromosome segregation. However, some Y chromosomes carry an ~115-kb extension (ePAR) via X-Y non-allelic homologous recombination. We found that ~0.77% of UK Biobank males carry ePAR, through 18 independent origins. Phenome-wide association links ePAR with elevated IGF-1 levels and musculoskeletal phenotypes.

Poriswanish et al., 2025, The American Journal of Human Genetics *112*, 927–939
April 3, 2025

© 2025 The Author(s). Published by Elsevier Inc. on behalf of American Society of Human Genetics.

https://doi.org/10.1016/j.ajhg.2025.01.026

Multiple origins and phenotypic implications of an extended human pseudoautosomal region shown by analysis of the UK Biobank

Nitikorn Poriswanish,[1,2,5] James Eales,[3,5] Xiaoguang Xu,[3] David Scannali,[3] Rita Neumann,[1] Jon H. Wetton,[1] Maciej Tomaszewski,[3,4] Mark A. Jobling,[1,*] and Celia A. May[1,*]

Summary

The 2.7-Mb major pseudoautosomal region (PAR1) on the short arms of the human X and Y chromosomes plays a critical role in meiotic sex chromosome segregation and male fertility and has been regarded as evolutionarily stable. However, some European Y chromosomes belonging to Y haplogroups (Y-Hgs) R1b and I2a carry an ~115-kb extension (ePAR [extended PAR]) arising from X-Y non-allelic homologous recombination (NAHR). To investigate the diversity, history, and dynamics of ePAR formation, we screened for its presence, and that of the predicted reciprocal X chromosome deletion, among ~218,300 46,XY males of the UK Biobank (UKB), a cohort associated with longitudinal clinical data. The UKB incidence of ePAR is ~0.77%, and that of the deletion is ~0.02%. We found that Y-Hg I2a sub-lineages accounted for nearly 90% of ePAR cases but, by Y haplotyping and breakpoint sequencing, determined that, in total, there have been at least 18 independent ePAR origins, associated with nine different Y-Hgs. We found examples of ePAR linked to Y-Hg K among men of self-declared Pakistani ancestry and Y-Hg E1, typical of men with African ancestry, showing that ePAR is not restricted to Europeans. ePAR formation is likely random, with high frequencies in some Y-Hgs arising through drift and male-mediated expansions. Sequencing recombination junction fragments identified likely reciprocal events, and the heterogeneity of ePAR and X-deletion junctions highlighted the recurrent nature of the NAHR events. A phenome-wide association study revealed an association between ePAR and elevated levels of circulating IGF-1 as well as musculoskeletal phenotypes.

Introduction

The human X and Y chromosomes undergo obligate crossing over during male meiosis within a specialized domain of strict homology, the ~2.7-Mb pseudoautosomal region 1 (PAR1; Figure 1A), which lies at the tips of the short arms.[1,2] The key role of this region in sex-chromosomal recombination[3] and the correct segregation of X and Y into sperm[4] suggests that its conservation is important, and it has been regarded as evolutionarily stable throughout most of the taxonomic span of the catarrhine primates.[5,6] However, the discovery of polymorphism in the position of the human PAR1 boundary[7] challenged this: an analysis of aCGH (array comparative genomic hybridization) data in ~4,300 Belgian and French individuals revealed a set of 15 pedigrees that carried an extended PAR1 (ePAR), showing that the PAR1 boundary is not static among humans. The extended region of homology between an X chromosome and ePAR-carrying Y is expected to support sex-chromosomal crossover, and we confirmed this by analyzing crossover directly in the sperm of males carrying an ePAR.[8]

Molecular analysis of males carrying an ePAR[7] indicated that it arose from non-allelic homologous recombination (NAHR) between directly repeated ~550-bp LTR6B elements: one of these lies within PAR1, ~4.8 kb distal to the boundary, while the other lies in normally X-specific DNA, ~115 kb proximal to the boundary (Figure 1B). When ectopic crossover occurs between these elements, two products can arise—a Y chromosome carrying a 115-kb extension of perfect pseudoautosomal XY homology, representing the ePAR, and a reciprocal internally shortened X chromosome that has lost the ~115 kb of DNA between the two LTR6B repeat elements. Such a deleted X chromosome was identified via the aCGH study.[7] Notably, the deleted X also lacks functional copies of two genes, *XG* and *GYG2*, though a clinical investigation of two families segregating the deletion suggested that phenotypic effects are not severe.[9]

Because the generation of an ePAR occurs on a particular Y chromosome, it is expected to be associated with a particular lineage of the male-specific region of the Y (MSY) that could be assigned to a Y haplogroup (Y-Hg) through the typing of MSY single-nucleotide polymorphisms (SNPs). In the original study,[7] most of the ePAR-carrying pedigrees discovered carried Y chromosomes belonging to Y-Hg I2a, but in two pedigrees, the ePAR Y chromosomes belonged to a distantly related haplogroup, R1b. This demonstrated that the ePAR had been generated independently at least twice—a finding not unexpected for NAHR-mediated events, which tend to be recurrent.[10]

[1]Department of Genetics, Genomics and Cancer Sciences, University of Leicester, Leicester, UK; [2]Department of Forensic Medicine, Faculty of Medicine Siriraj Hospital, Mahidol University, Bangkok, Thailand; [3]Division of Cardiovascular Sciences, Faculty of Biology, Medicine and Health, University of Manchester, Manchester, UK; [4]Manchester Academic Health Science Centre, Manchester University NHS Foundation Trust Manchester, Manchester, UK
[5]These authors contributed equally
*Correspondence: maj4@le.ac.uk (M.A.J.), cam5@le.ac.uk (C.A.M.)
https://doi.org/10.1016/j.ajhg.2025.01.026.
© 2025 The Author(s). Published by Elsevier Inc. on behalf of American Society of Human Genetics.
This is an open access article under the CC BY license (http://creativecommons.org/licenses/by/4.0/).

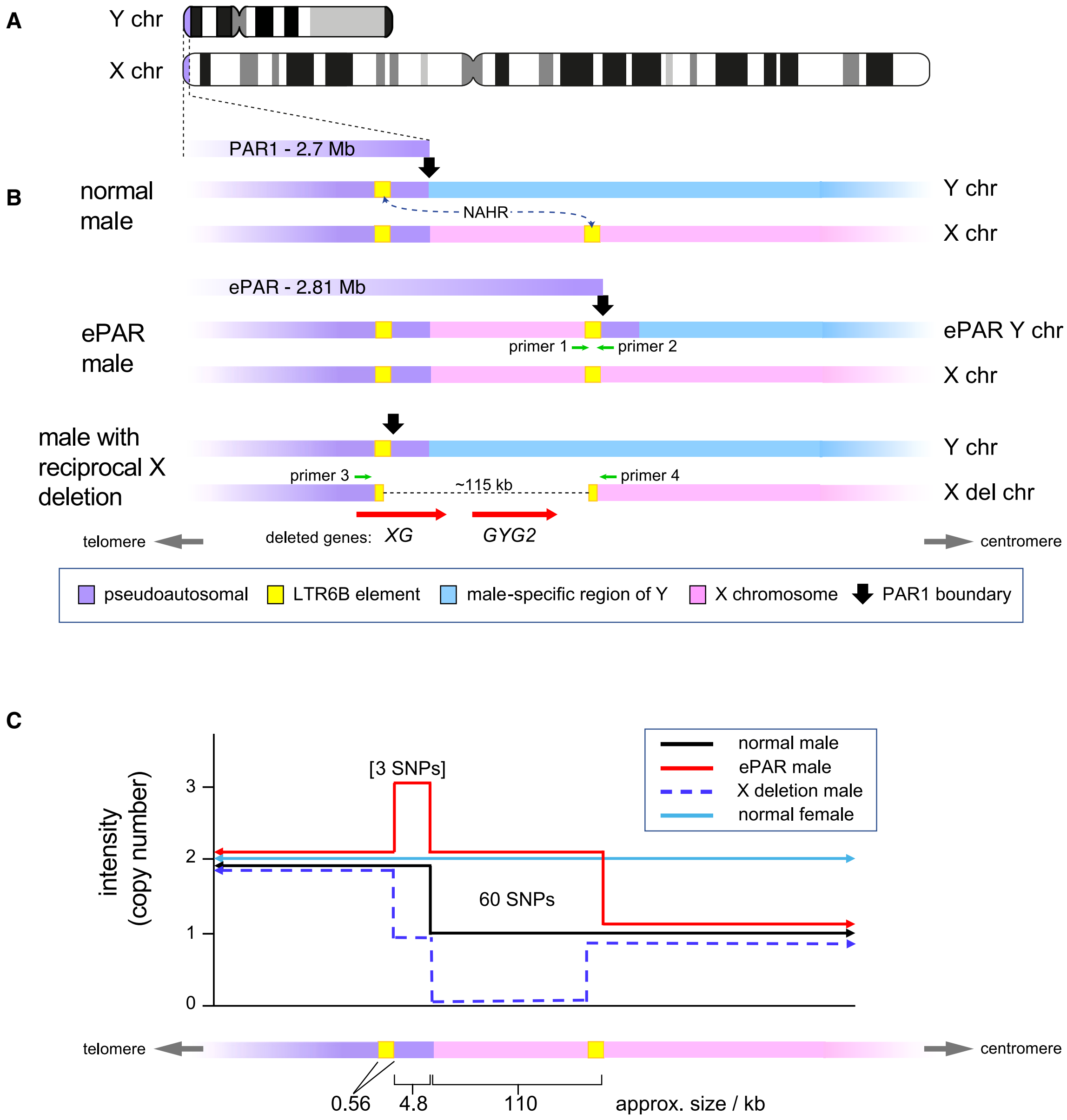

Figure 1. Rationale adopted to identify cases of ePAR and the predicted reciprocal deletions from UKB SNP microarray data
(A) Idiograms of the X and Y chromosomes, showing the position of PAR1.
(B) Structures (not to scale) of the X and Y chromosomes at the proximal ends of PAR1 as carried by a normal 46,XY male (top), an ePAR Y chromosome carrier (middle), and a male with the expected reciprocal deletion of the X chromosome (bottom). The ePAR rearrangements are caused by NAHR events between the PAR1-associated LTR6B element on a Y chromosome and the proximal LTR6B element on an X chromosome (indicated by the dotted arrow). Genes lying within the X chromosome deletion are shown with the direction of transcription indicated (red arrows). Green arrows indicate the positions of primers used to confirm the rearrangements (primer 1: ePARjunc-F, primer 2: ePARjunc-R, primer 3: FB, and primer 4: R2).
(C) Schematic representation of predicted SNP intensity data as a proxy for copy number over this region. ePAR carriers (red line) and males carrying the reciprocal X deletion (blue dashed line) are compared with normal 46,XY males (black line) and females (light blue line). The most distal portion of the X chromosome (chrX:2,776,959–2,890,559 [GRCh38.p12]) is expected to have a copy number of 1, 2, or 0 and the most proximal portion of the canonical PAR1 a copy number of 2, 3, or 1 for normal males, males with ePAR, and males with the reciprocal deletion, respectively. The Axiom array carries 60 informative SNPs across the region. The three SNPs falling in the interval predicted to be present in triplicate in males carrying ePAR were not evaluated (see the main text).

A clearer understanding of the diversity, history, and dynamics of ePAR formation could be obtained by studying ePAR-carrying Y chromosomes in a larger and more diverse cohort than that examined so far. A suitable resource for such analysis is UK Biobank (UKB),[11,12] a >20-year cohort study based on >500,000 UK individuals aged 40–69 years when recruited during 2006–2010, about half of whom declared themselves to be males.[11] As well as measuring extensive phenotypic and clinical data, DNA from each participant has been analyzed using the Biobank's second-generation dense genotyping chip (UKB Axiom Array) containing over 820,000 SNPs (http://www.ukbiobank.ac.

uk/scientists-3/uk-biobank-axiom-array/). These include >20,000 X chromosome SNPs (incorporating PAR1) and 813 Y-specific SNPs defining the main clades and some sub-branches of the MSY phylogeny, as reported earlier.[13]

The original identification of ePAR relied on the inference of a copy-number increase of an X chromosome segment in males based on SNP intensity information from a genome-wide SNP chip. A similar approach was taken here with the UKB cohort males, a sample about 50 times larger than the set screened previously[7] and which includes not only the "White British" census classification (88.5%) but also participants from the UK's minority ethnicities, including African, African-Caribbean, and South and East Asian individuals,[11] and is therefore likely to give a better overview of the distribution of ePAR among Y chromosomes from a wide range of haplogroups.

Here, we describe the screening of the UKB males for the presence of the ePAR and the reciprocal X chromosome deletion. We use a combination of haplotyping and recombination breakpoint sequence analysis to characterize these rearrangements and estimate the number of independent events giving rise to the ePAR. Finally, we ask if there are phenotypic consequences associated with these sex-chromosomal rearrangements.

Material and methods

Screening for ePAR and the reciprocal X chromosome deletion in UKB males was undertaken after excluding men showing an uneven balance of the two sex chromosomes (including sex-chromosomal aneuploidies) by assessing the dosage of 60 X-SNPs in Axiom array data within a 113-kb interval chrX:2,776,959–2,890,559 (GRCh38.p12) (corresponding to structural variants identified in dbVAR as nsv4041309 and nsv4040934 and in gnomAD[14] as DEL_X_184281 and DUP_X_52437). Normalized signal intensity for the ePAR X-SNPs was calculated as the median of deviation from the median in females. The SNP list for the UKB Axiom array was accessed at http://www.ukbiobank.ac.uk/scientists-3/uk-biobank-axiom-array/.

DNA samples were obtained and analyzed under UKB main application 32497 ("Numerical abnormalities and structural rearrangements of the Y and X chromosomes and predisposition to complex diseases"). This study was covered by the generic ethical approval for UKB studies from the NHS National Research Ethics Service (ref. 11/NW/0382).

A multiplex PCR encompassing nine haplogroup-defining SNPs within Y-Hg I2a (Table S1) was carried out as previously described.[8] Y-STR (short tandem repeat) haplotypes were generated using Promega's PowerPlex Y23 System (PPY23) as per the manufacturer's guidelines, with the products run on an Applied Biosystems 3130xl Genetic Analyzer and analyzed by GeneMapper ID v.4.0 software. Y-Hgs were then predicted using the batch capability function in the desktop version of NevGen Genealogy Tools Software v.1.1 (http://www.nevgen.org/, with the 10/15/2019 NevGenGeneralPredictor_23_STR.dat data file). This software compares a queried Y-STR haplotype against a reference dataset of haplotypes associated with known haplogroups, employing a Bayesian allele frequency approach based on that of Athey[15] and considering pairwise allele correlations between different Y-STRs in estimating haplogroup probabilities. Median-joining networks of the haplotypes were generated using Network 5 together with Network Publisher software (http://www.fluxus-engineering.com/).[16] Dating of Y-STR clusters was performed using the ASD (average square distance) method.[17]

Amplification of an 848-bp PCR fragment using primers spanning the ePAR junction (from the distal X-specific LTR6B to the proximal PAR1-specific LTR6B; Figure 1B) was used to confirm the presence of the ePAR, as described previously.[8] The assay coamplifies a 1,551-bp control fragment from the Y chromosome *SRY* gene to confirm successful PCR in the absence of an ePAR fragment (Figure S1). Primer sequences were ePARjunc-F (5′-TGGCAATGTTACTGGAGACG-3′), ePARjunc-R (5′-CAAGGAGTCTGCTGGAAGTC-3′), SRY-F (5′-GGGGTCCCGAGATTTATGTT-3′), and SRY-R (5′-GCTAGAACAAGTTACCCCTC-3′). Candidate reciprocal deletions were amplified using primers FB (5′-CCCTAGAACCAGAAGCAATG-3′) and R2 (5′- CAGTGTTCTTGGACAGAGGC-3′), which in a normal X chromosome lie ~116 kb apart (Figure 1B), but in an X-deletion chromosome generate a junction product of ~1.96 kb. PCR products were gel purified using the Zymoclean Gel DNA Recovery kit (Zymo Research) per the manufacturer's recommendations and then Sanger sequenced using BigDye Terminator v.3.1 chemistry (Applied Biosystems). In addition to primers FB and R2, the reciprocal deletion breakpoints were sequenced with primer N4 (5′-CCCCGAATTCTTTCTTGCCG-3′). The generated sequences are provided in Data S1.

Amplicons containing the Y-Hg K2a-defining SNPs were generated using Y28299F (5′-GAGCCACTGCACCCGAGA-3′) with Y28299R (5′-CTTCTGCATGATACATGAGGCA-3′) and Y28300F (5′-GATTGTTTTGGGCTCAGGTGT-3′) with Y28300R (5′-CTGACATTTAGAATGGTGCCT-3′). The gel-purified products were Sanger sequenced as above with nested primers (Y28299F2 5′-TCTTTGTAGGAGAGTTGGCT-3′; Y28299R2 5′-GTTCAAATCATACTAGTGGGTC-3′; Y28300F2 5′ GACTGAACCCGGAGCTTTGA-3′; and Y28300R2 5′- TGGAGGTTGAAAGCGGAGAC-3′).

Phenome-wide association study (PheWAS) analysis to investigate possible phenotypic effects of the ePAR (outlined in Figure 5) employed an extension of the R package PEACOK,[18] which integrates Firth logistic regression to address imbalanced data for binary outcomes. The package tests the association of genetic variants (here, carriage of ePAR) with the heterogeneous collection of all binary and continuous phenotypic variables in the UKB. Automated phenome scanning is enabled using a rule-based algorithm to decide on the appropriate model for testing each phenotype. The PheWAS for the reciprocal X chromosome deletions in male hemizygotes and female carriers followed the same approach.

Results

Screening for ePAR among UKB males

Of the >20,000 X chromosome SNPs genotyped by the UKB Axiom array, 60 were identified based on their coordinates to lie in the distal segment known to form part of ePAR[7,8] (see Table S2). The dosage, and therefore the fluorescent intensity, of these X-SNPs is predicted to be doubled in males who carry an ePAR compared to those who do not and to be equivalent to values seen in normal females, as shown in Figure 1C. Given the known underlying rearrangement associated with reported cases of ePARs,

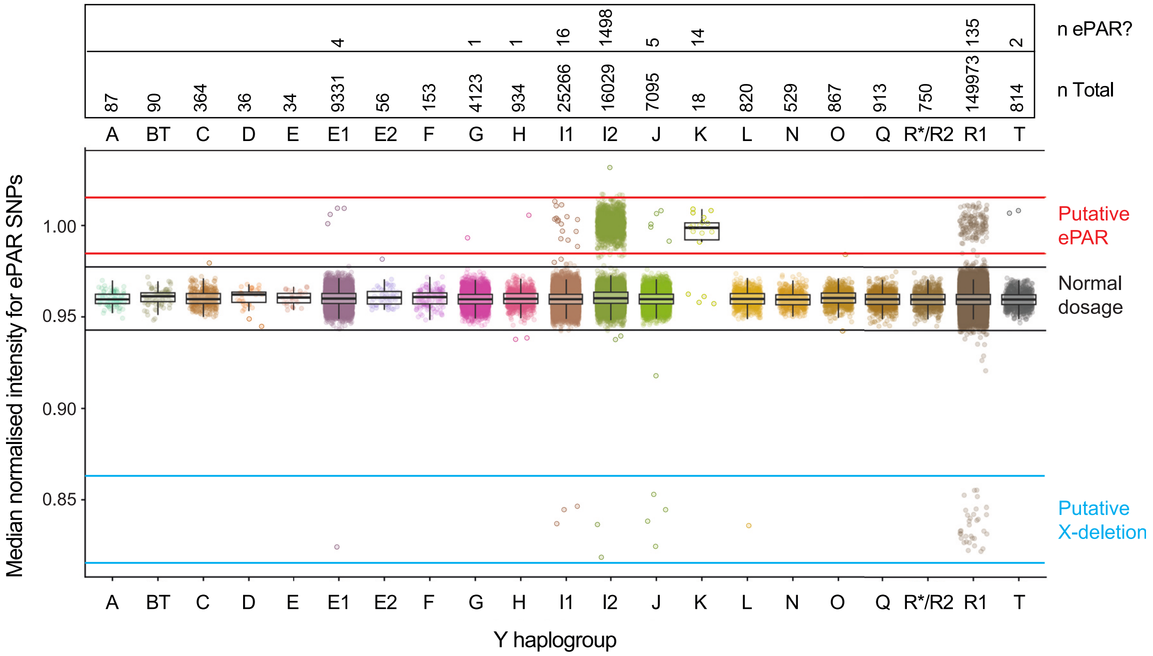

Figure 2. Median normalized intensity data for the ePAR-informative SNPs among UK Biobank 46,XY males categorized by Y-Hg
The majority of men (total $n = 218{,}282$) showed median normalized intensities for the ePAR-relevant SNPs consistent with them being present in single copy, as indicated by the pair of horizontal solid black lines (95% CI for haploid X). By contrast, 1,676 men exhibited intensities for these X chromosome SNPs in the range expected for normal females, as indicated by the pair of horizontal solid red lines (95% CI for diploid X), suggesting that they are putative ePAR carriers. The number of men and the number of putative ePAR carriers per Y-Hg are given above the plot. Some men were also observed to have intensities for these SNPs considerably lower than expected for a haploid X chromosome (between the horizontal blue lines); these are putative carriers of the predicted reciprocal NAHR product that results in a deletion in this interval. See Figure S2 for intensity data for females.

three proximal X-SNPs were also expected to be present in a triple dose, but given this very small number and the natural variation in SNP-chip intensity data, we did not attempt to assess dosage in this interval.

On this basis, screening for ePARs was initially carried out on 223,605 UKB men, representing ~98% of the entire sample of males in the cohort. We first filtered out males showing an uneven balance of the two sex chromosomes (including sex-chromosomal aneuploidies) by determining the median log2 ratio of markers on the X and Y chromosomes excluding all PAR-associated markers. We then attempted to determine Y-Hgs using the yHaplo software,[19] based on the presence of ancestral or derived states of 172 of the 813 Y-SNPs on the array, checked against a reference MSY phylogeny (Table S3). A definitive designation of a total of 100 distinct haplogroups was achieved across a total of 218,282 men (Table S4). In these individuals, signal intensity for the ePAR-informative X-SNPs was calculated as the median of deviation from the median in females, to give a normalized median intensity. In this way, a total of 1,676 or ~0.77% (approximately 1 in 130) of the UKB males were identified as putative ePAR carriers (Figure 2). The screening approach also identified 44 males as putative carriers of the reciprocal X chromosome deletion. Surveying 264,300 UKB females revealed 137 who were apparently heterozygous for the same deletion and no apparent homozygous deletions (Figure S2). Jointly considering the frequencies of the deletion in hemizygous males and heterozygous females[20] shows that the X-deletion allele is in Hardy-Weinberg equilibrium in both sexes (Table S5), suggesting that any effect on fitness is minor.

We next considered the association of the apparent ePARs with Y-Hg. The majority (1,498 males, i.e., ~89%) were associated with Y-Hg I2 (Figure 2), carried by just ~7.4% of UKB males ($p < 0.00001$; chi-squared test), showing that ePAR frequency does not mirror Y-Hg frequency. This also supports the previous findings of ePAR associated with I2a[7] and indicates that our ePAR ascertainment approach was valid. Further verification was achieved by positive results with an ePAR junction-specific PCR (Figure S1)[8] on a randomly chosen subset of 102 (~7%) of the Y-Hg I2a carriers. In addition to Y-Hg I2a, the SNP intensity screen indicated that ePAR may have also arisen on a further eight broadly defined Y-Hgs, including R1 (Figure 2), the only other haplogroup previously reported[7] to be associated with ePAR. Of the 178 non-I2a putative ePAR carriers identified in the UKB, 87 were also available for analysis using the junction-specific PCR. Of these, 39 males (~45%) from four broad Y-Hgs (E1, I1, K, and R1b) gave positive results. The remaining 48 (0.022% of the UKB males screened) could be carriers of larger extended segments than the canonical ePAR, but there is no evidence of haplogroup clustering, which

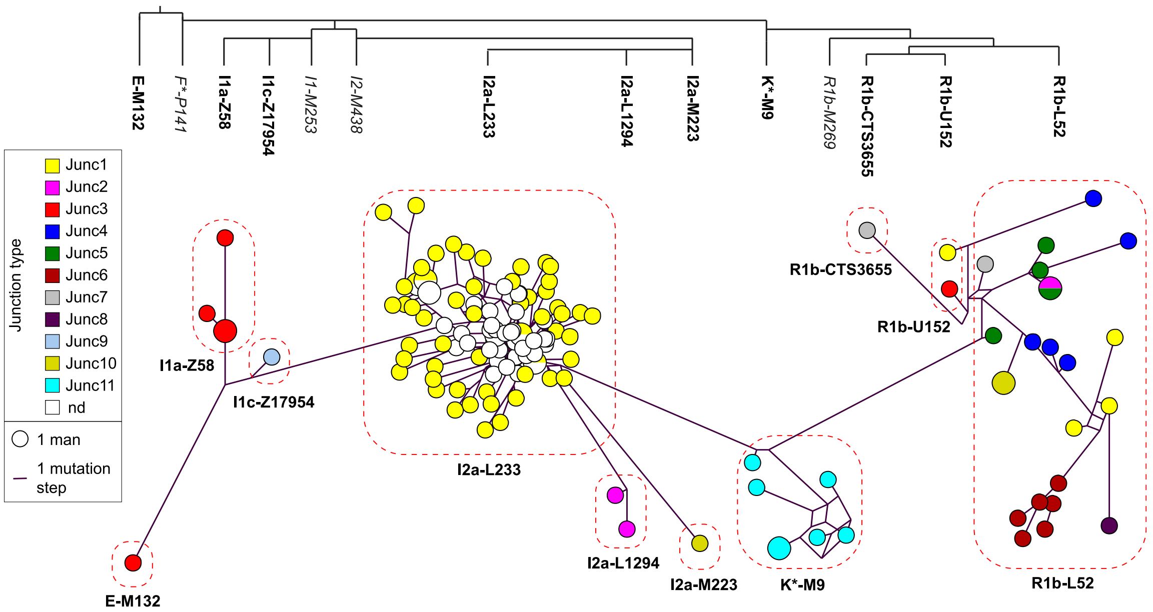

Figure 3. Distribution of ePAR junction types with respect to Y-STR haplotypes and inferred Y-Hgs
The phylogeny (top) shows the relationships between relevant haplogroups; ePAR-containing Y-Hgs are in bold, and some other haplogroups are shown (italics) for orientation purposes. On the bottom is a median-joining network generated from Y-STR haplotypes for all ePAR-carrying males indicated with respect to both Y-Hg (dashed shapes) and ePAR junction type (node colors, as indicated in the key, left). The sizes of the nodes are proportional to the number of individuals typed and branch lengths to the number of mutational differences between linked nodes, as shown in the key. nd, not determined.

might suggest founder mutations.[21] Alternatively, they may carry supernumerary X chromosome segments translocated to other parts of the genome. These were not analyzed further.

Diversity of Y-Hgs carrying the ePAR

Based on the array Y-SNP data and junction PCR results, confirmed ePARs belong to eight Y-Hgs (E1a-M132, I1-M253, I2a-M223, I2a-M438, K*-M9, R1b-CTS3655, R1b-L52, and R1b-U152). To confirm Y-SNP-based haplogroup calls and further refine haplogroup designations, the 141 DNA samples (102 Y-Hg I2a, plus 39 males carrying other haplogroups) were typed for 23 Y-STRs using the PPY23 kit and haplogroups were predicted from the resulting STR haplotypes (Table S6) using NevGen Genealogy Tools software (http://www.nevgen.org/). These predictions resolved three Y-Hgs to more derived sub-lineages: I2a contained chromosomes predicted as I2a-L233 and I2a-L1294 in addition to the SNP-confirmed I2a-M223; I1 comprised two sub-haplogroups, I1a-Z58 and I1c-Z17954; and R1b comprised three sub-haplogroups in total, R1b-L52, R1b-U152, and R1b-CTS3655. However, prediction was unable to reliably categorize samples belonging to the haplogroup defined by the Axiom array Y-SNPs as K*-M9 ($n = 7$), possibly because it is underrepresented in the reference datasets used by the NevGen predictor. Apart from this unpredicted haplogroup, the remainder showed 100% concordance of Y-Hg identification between the two approaches. In total, therefore, 10 Y-Hgs were found to be associated with ePAR-carrying chromosomes. Given the phylogenetic relationships of these lineages based on a consideration of the ISOGG tree (https://isogg.org/tree/), this represents a minimum of 10 independent ePAR occurrences.

Since the Y-Hg most often associated with ePAR is I2a, and we have previously found independently sampled I2a ePARs to be identical by descent,[8] we explored this further in the UKB cohort. The predicted I2a sub-haplogroups were verified by Y-SNP typing in a subset of 22 of the UKB men using a custom-designed SNaPshot assay (Table S1),[8] confirming that there are indeed three independent ePAR lineages within Y-Hg I2a. All males whose Y chromosomes were predicted to belong to Y-Hg I2a-L233 had been identified as putative ePAR carriers from the UKB Axiom array, and the majority were found to carry similar Y-STR profiles, indicative of a founder effect prior to the expansion of the L233 SNP lineage. A median-joining network constructed using Y-STR haplotypes for all confirmed ePAR chromosomes is shown in Figure 3.

ePAR junction diversity among UKB males

To investigate the history of ePAR formation, we explored the sequence diversity of the NAHR junctions. Sanger sequencing was applied to a total of 97 ePAR LTR6B junction fragments derived by PCR from the UKB samples (see Data S1). These consisted of a subset of 55 samples from Y-Hg I2a-L233, chosen to maximize diversity by taking all the outlying nodes and a few from the inner core of the Y-STR-based network (Figure 3), plus the other ePAR-carrying samples not associated with Y-Hg I2a-L233: I2a-L1294 ($n = 2$), I2a-M223 ($n = 1$), I1a-Z58 ($n = 4$), I1c-Z17954 ($n = 1$), R1b-L52 ($n = 23$), R1b-U152 ($n = 2$), R1b-CTS3655 ($n = 1$), K*-M9 ($n = 7$), and E1a-M132 ($n = 1$).

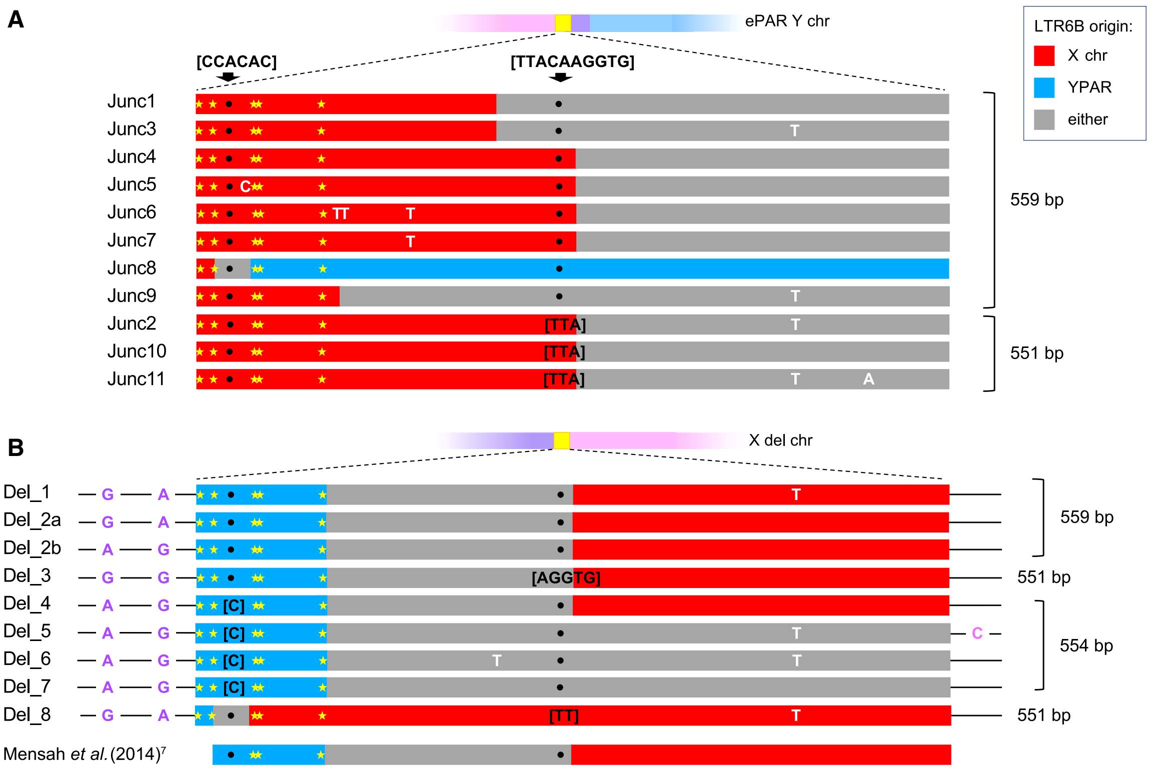

Figure 4. Sequence structures of LTR6B junctions in derived Y and X chromosomes

(A) Schematic of the ePAR Y chromosome as in Figure 1 together with the structures of the 11 characterized UKB ePAR LTR6B junctions. Fixed differences between the X-LTR6B and YPAR-LTR6B based on 1000 Genomes Project data[22] are indicated by the yellow stars. Junction fragments vary in overall size as a consequence of two variable length regions, as indicated by the arrows above the structures ([CCACAC] at the distal site and [TTACAAGGTG] at the more proximal site). These are the longest variants at each site and are represented by black dots within the structures; alternative length variants are shown in the corresponding positions (black text). Other distinguishing sites of variation are shown in white text. Where an inference can be made as to the origin of a particular segment of each junctional LTR6B element, it is shown as a red (X) or blue (YPAR) bar and gray otherwise. The overall length of junction sequence is indicated on the right.

(B) Schematic of the X-deletion chromosomes and structures of the nine characterized UKB X-deletion junctions are shown as in (A) together with flanking SNP haplotype information from rs311159 and rs2109378 associated with YPAR (purple) and rs211658 from the X chromosome (pink) (positions not to scale). The partial structure of the X-deletion junction previously described[7] is shown at the bottom. Full details of all the sites of variation used to determine these junction structures can be found in Table S7.

Eleven distinct LTR6B junction sequences were identified, including the two types (known as Junc1 and -2) previously described,[7] and are indicated by color coding on the network in Figure 3. We name the nine newly described junction sequences Junc3–Junc11 (Figure 4; Table S7). The most commonly observed ePAR junction sequence, carried by 59 of the 97 UKB males, was the 559-bp Junc1-type LTR6B element previously found to be associated with both I-P37.2* and R-P312*Y chromosomes.[7] Consistent with this, Junc1 was predominantly found on I2a-L233 Y chromosomes (55 of the 59), on one of the two R1b-U152 chromosomes, and also on three of the more ancestral Y-Hg R1b-L52 among the UKB males. The shorter 551-bp Junc2-type LTR6B previously found to be associated with I-P37.2* was found in just three of the 97 UKB males, two belonging to I2a-L1294, another derived sub-lineage of I2a-P37.2, and the other to R1b-L52. The novel 559-bp Junc3 was found to be associated with Y-Hg I1a and E1a-M132, and the novel 551-bp Junc11 was associated with the seven Y-Hg K*-M9 chromosomes. Six of the remaining seven new junction types (Junc4–Junc8, all 559 bp long, and Junc10, 551 bp long) were found among R1b-L52 Y chromosomes (reflecting the fact that this is the most common sub-haplogroup observed among UKB males, at 42.6%), showing that these ePARs have diverse origins. The remaining 559-bp junction observed only once, Junc9, was associated with the single I1c Y chromosome.

Taking the evidence from associated haplogroups and junction sequences together, we estimate a minimum of 18 independent events among the UKB males, underlining the recurrent nature of ePAR formation (Figure 3). The rate of the process should be deducible given knowledge of the number of generations within the phylogeny relating all analyzed chromosomes,[23,24] but this number is difficult to estimate for the UKB dataset. The 1000 Genomes Project's diverse 1,244 MSY sequences[19] contain 60,555 total SNPs, with a mean number of SNPs to each tip clade of ~1,300. Assuming a time to most recent common ancestor (TMRCA) of 190,000 years[19] and a generation time of 31

years,[25] this gives a total time of ~283,000 generations. We analyzed ~218,000 UKB chromosomes in which all major clades are represented (Table S4), albeit most at very low frequency, while a few (e.g., within Y-Hgs R1b, R1a, I1, and I2) are common and must contain closely related Y chromosomes. The value of 283,000 generations is therefore likely to be an overestimate here but gives an ePAR formation rate of $\sim 6.5 \times 10^{-5}$ per generation, compatible with rates for a number of NAHR hotspots determined directly in sperm DNA.[26]

Junction typing also provides additional evidence that ePAR can support recombination along its entire length. Two R1b-L52 donors with Junc5 and Junc2 differing by a C/T SNP (position 39; Table S7) and the A/ACAAGGTG insertion or deletion (indel; position 269) share the same PPY23 Y-STR haplotype (shown as a bicolored node in Figure 3). Profiling both donors with a further five highly polymorphic Y-STRs (DYS449, DYS460, DYS518, DYS627, and DYF387S1) revealed just a single repeat difference at one of the additional loci between them, confirming that these men must share very recent ancestry. Two other R1b-L52 donors with very similar PPY23 haplotypes also carry Junc5, suggesting it is the ancestral state for this ePAR lineage, whereas Junc2 is the sequence of the reference X chromosome LTR6B element. The most parsimonious explanation for the observed data is that an X/ePAR recombination event occurred recently within 290 bp of the extended boundary produced by ePAR in this lineage.

We attempted to infer the most likely location of the NAHR crossover events in light of reported polymorphisms from the 1000 Genomes Project[22] to alleviate bias in interpretation when simply comparing the two genome reference versions of the LTR6B elements (Figure S3). Five apparently fixed differences were noted within the first 100 bp of the two elements, allowing the crossover point for one of these recombinant LTR6Bs to be localized to a ~30-bp interval within 45 bp of the 5′ end (Junc8 in Figure 4A). All other crossover points were inferred to be proximal to these fixed sites, but it was impossible to determine definitive exchange points because reference and alternative alleles were shared for the 19 remaining variant positions in the two LTR6Bs (see also Table S7). Indeed, we noted a non-random pattern of variation between these two parental elements, whereby the minor alleles of the X-LTR6B tend to be identical to the major alleles of the Y-PAR copy, but not vice versa ($p = 0.02$; one-tailed Fisher's exact test). This would be consistent with subsequent "homologous" exchanges between ePAR elements and the X-specific LTR6B in ePAR carriers, though curiously, the most proximal fixed difference lies among these sites. It is also noteworthy that among these ePAR junctions, the position corresponding to rs2316283/rs2534626 in each of the elements is more frequently a G than a T, despite the latter being reported in the 1000 Genomes Project dataset[22] as the major allele in the X-specific LTR6B and fixed for the Y-PAR copy (minor-allele frequencies [MAFs] = 0.96 and 1.00, respectively; $n = 3{,}775$). Therefore, attempts to interpret the junction crossover breakpoints using a modified reference for each LTR6B element, based on major alleles alone, also failed to identify simple definitive exchange points for the majority of ePAR junction structures.

Characterization of the X chromosome reciprocal deletions

Of the 44 males identified via SNP intensity data from the UKB Axiom array as carrying the putative reciprocal X chromosome deletion, 24 were available for further characterization. Primers expected to give an ~1.96-kb product encompassing the LTR6B element in deletion-bearing X chromosomes, but otherwise located ~116 kb apart in the assembled human genome, were used to generate templates for sequencing (Data S1). Eight different LTR6B deletion breakpoint configurations were identified, with the recombinant elements being 551, 554, or 559 bp in length and the most common type seen seven times among the 24 sampled X chromosomes (Figure 4B; Table S7). One, Del_8, shared the ~30-bp inferred exchange interval with Junc8-type ePAR, suggesting that these might represent the reciprocal products of an NAHR event. For four of the deletion-associated LTR6B elements, breakpoints could be localized within an ~187-bp interval delineated by the most proximal fixed difference and the SNP rs12843082 located in the center of the element. However, as with the ePAR junctions, it was not possible to definitively localize the exchange points for the remainder of the deletion-associated elements, given the variation known to exist within each of the parental LTR6B elements. Although the single deletion previously reported[7] was only partially sequenced in comparison, over this region, it was found to match the second most common LTR6B deletion type, Del_2, among those characterized here from the UKB. In fact, in our approach, a total of ~1.3 kb of flanking sequence was also obtained, and two sites of variation were noted within the 525 bp of the Y-PAR sequence distal to the element (SNPs rs311159 and rs2109378), together with a single X chromosome site 269 bp proximal to the element (rs211658). Taking this into account, a total of nine different deletion-bearing X chromosomes were identified among these 24 males (Figure 4B; Table S7), again a testament to the recurrent nature of the events giving rise to both ePAR and these X chromosome deletions.

Founder effects among ePAR Y chromosomes

The LTR6B elements driving ePAR formation lie in PAR1 and X-specific DNA, with no involvement of MSY sequences. This implies that ePARs should originate randomly with respect to Y-Hgs; the variation in ePAR frequency among haplogroups is then due to subsequent drift and possible male-mediated expansions affecting some lineages.[27]

Despite the high level of recurrence, it is clear that most ePAR-confirmed carriers not only possess the same Y-Hg,

I2a, borne by ~7.4% of UKB males but that they represent a sizable proportion of men with this haplogroup (~9.3%). The Y-STR genotyping, haplogroup prediction, and subsequent SNaPshot sub-haplogroup typing indicate that the majority of these ePARs are very closely related, belonging to the I2a-L233 sub-lineage, and are likely to all carry the Junc1 NAHR junction sequence. Using 97 of these UKB-confirmed ePAR carriers with complete 23-locus Y-STR profiles, we obtained a mean estimate of the TMRCA of around 2,000 years before present (ybp) (2,007 ± 403 ybp [using all Y-STRs], 2,040 ± 1,202 ybp [using the 11 slower-mutating Y-STRs only], or 1,999 ± 397 ybp [using the 12 faster-mutating Y-STRs]). At the time of the common ancestor, the UK population was approximately 2–3 million[28] and by 2022 had increased to 67.6 million (Office of National Statistics). This implies that the proportion of men carrying the I2a-L233 Junc1 ePAR lineage has increased approximately 6,700-fold during this period and suggests that there has not been strong selection against the ePAR1. Publicly available MSY sequence data can also be used to evaluate the history of the lineage (Figure S4). Based on a phylogeny of sequences from DNA donors to a direct-to-consumer testing company, FamilyTreeDNA (https://www.familytreedna.com/public/y-dna-haplotree/I), the lineage contains a striking star-like expansion. Dating based on SNP accumulation gives a TMRCA that agrees well with our STR-based estimate (1,980 ybp [95% confidence interval [CI]: 1,680–2,332 ybp]).

A further potential founder effect was highlighted by the cluster of Junc11 males within the poorly resolved Y-Hg K*-M9 (Figure 3). These seven were the only samples available from a total of 14 putative ePAR carriers noted among just 18 UKB men (i.e., ~78%) assigned to this Y-Hg. As the Axiom array did not resolve Y-Hg K*-M9 sub-lineages, we examined the 1000 Genomes Project dataset[22] for males who might also be classified as K*-M9 if typed with the same SNP array. One male, HG03742, a member of the Indian Telugu in the UK (ITU) population, showed evidence of the expected increase in read depth over the ePAR interval and was known to carry the rarely observed Y-Hg K2a (Figure S5).[19] Sanger sequencing of two SNPs that define this lineage (rs767631170 and rs776080867, corresponding to K2a-Y28299 and K2a-Y28300) showed that all seven characterized UKB Junc11 males were indeed derived for both of these markers. These UKB carriers were of self-declared Pakistani ancestry, indicating the presence of an ePAR cluster within South Asia. In support of this, we obtained TMRCAs of 1,980 ± 398 (based on all 23 STRs), 2,417 ± 1,423 (11 slow mutators), and 1,870 ± 371 (12 faster mutators) ybp, indicating that the founding event occurred prior to substantial migration to the UK from the sub-continent.

In contrast to the Y-Hg I2a-L233 and K2a clusters, the remaining characterized ePARs occur within Y-Hgs that are common within the UKB dataset yet comprise only a small fraction of men carrying each of these broad Y chromosome types. For example, four putative ePARs were noted among 9,331 E1 men (0.04%), 16 among 25,266 I1 men (0.06%), and 135 among 149,973 R1b men (0.09%) (Figure 2), all significantly lower proportions than expected given the haplogroup frequencies ($p < 0.00001$; chi-squared test). As noted previously, the diverse junction types seen among some of these Y-Hgs indicate that these low frequencies are generated through independent events.

Phenotypic implications of the ePAR rearrangements

Given the extensive phenotypic and clinical data associated with the UKB cohort and the established role of sex chromosomes in human health and susceptibility to different common disorders,[29–31] we looked for ePAR-phenotype associations in a PheWAS. We first compared all 1,319 ePAR carriers to the 154,032 non-ePAR carriers (Figure 5; Table S8) and identified two of the 194 quantitative and seven of the 3,071 binary UKB phenotypes tested as being significantly associated with ePAR (false discovery rate [FDR] < 0.05). We then restricted our analyses to the 1,294 White British Y-Hg I2 ePAR carriers and compared these against all 153,896 non-males not carrying ePARs and then solely against the 1,663 non-ePAR Y-Hg I2 White British males to control for both ancestry and any factors relating to variation in other parts of the Y chromosome. One of the quantitative traits (mean corpuscular volume) was not retained in this comparison (Table S8). However, the other, elevated levels of circulating IGF-1 among ePAR carriers were consistently noted (Figure S6), even when extreme outliers were excluded (data not shown). The significantly associated binary phenotypes in the two comparisons are similar (Table S8). Weaker associations were also consistently found with the musculoskeletal system and bone health. We used the number of children fathered (data field 2405) as a proxy for male fertility but found no association (beta: −0.04, $p = 0.19$ for I2 ePAR vs. I2 non-ePAR; beta: −0.034, $p = 0.20$ for I2 ePAR vs. all non-ePAR).

Sample sizes of heterozygous female and hemizygous male carriers of the reciprocal X chromosome deletion are smaller than those of the ePAR, and this limits the power to detect any phenotypic consequences. Indeed, PheWAS analysis of females and males yields no associations for either continuous or binary traits that retain significance following correction for multiple testing (Table S9).

Discussion

This study underlines the dynamic nature of the human PAR1 and its boundary. By screening over 218,000 men in the UKB, we found that the incidence of the ePAR, a NAHR-mediated rearrangement between LTR6B elements that extends PAR1 and shifts the pseudoautosomal boundary some 115 kb proximally, is ~0.77%. Consistent with the original description of ePAR identified in a 50-fold

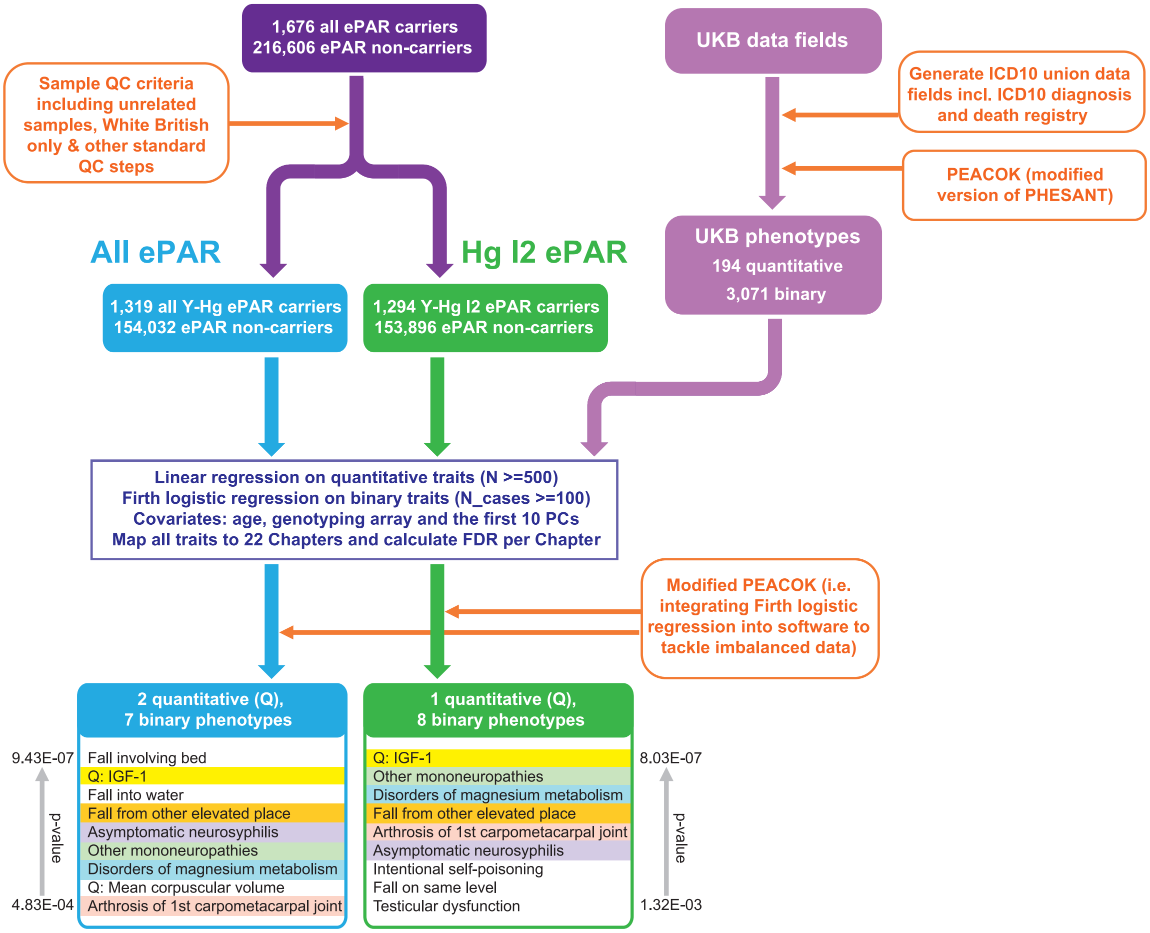

Figure 5. Summary of ePAR PheWAS scheme and results
Details of the associated phenotypes and *p* values are given in Table S8. ICD10, 10th revision of the International Statistical Classification of Diseases and Related Health Problems; PC, principal component; FDR, false discovery rate; PHESANT, Phenome Scan Analysis Tool.[32]

smaller dataset of French and Belgian families,[7] we find that nearly 90% of cases are associated with a particular Y chromosome lineage, Y-Hg I2a-L233, and share the same specific ePAR junction structures, demonstrating a founder effect. Using a suite of highly informative Y-STRs, we have been able to determine the TMRCA more accurately at ∼2,000 ybp and infer that this lineage has since increased by ∼6,700-fold, suggesting there has not been strong selection against the extended pseudoautosomal region.

In this study, ePAR was associated with Y-Hg R1b, as previously noted,[7,8] but it was also found in association with other Y lineages. Notably, ePAR is carried by Y-Hg K men of self-declared Pakistani ancestry and Y-Hg E1a-M132, typical of West Africa and the Caribbean. By also interpreting the ePAR junction sequences, we inferred that a minimum of 18 separate events are captured by this large cohort. This indicates that the recurrence of NAHR to generate the ePAR occurs more frequently than hitherto recognized and is not restricted to Europeans as previous studies suggested.[7,8] The dynamic nature of PAR1, as demonstrated here, is further supported by recent evidence that the pseudoautosomal boundary has shifted location at least six times during primate evolution.[33]

The incidence among UKB men of the predicted reciprocal X deletion generated by the NAHR events was found to be ∼0.02%—considerably lower than the 0.7% carrying the ePAR. We expect the rates of formation of the two rearrangements to be similar, as they are the reciprocal products of the same NAHR event. However, ePAR is linked to the Y chromosome, which, due to its low effective population size, is particularly prone to drift. Further, some lineages (a possible candidate being Y-hg I2a-L233) can be influenced by past male-mediated expansions associated with social or economic circumstances in which a subset of males in a population had particularly high reproductive success.[27] The population frequency of the reciprocal X-deletion event is not influenced in this way, and we, therefore, do not necessarily expect the frequencies of the two rearrangements to be similar. Though the majority of males carrying X deletions carry Y-Hg R1b chromosomes, there is no causal link between the two since the X chromosome event is independently inherited—it simply reflects the fact that Y-Hg R1b is the most common

haplogroup among European men and those in the UKB (Table S4).

The low observed frequency of X deletions could, in principle, also be influenced by negative selection against hemizygous males. The fact that the deletion is in Hardy-Weinberg equilibrium (Table S5) argues against this idea. Further, given the extensive phenotypic data associated with the UKB, it was possible to carry out PheWASs among the observed male (hemizygous) and female (heterozygous) carriers of deletions. These revealed no statistically significant effects in the small sample sizes available (Table S9).

Males carrying the X deletion are expected to produce no protein products for two genes: *GYG2*, encoding glycogenin-2 (GLN2), the predominant glycogenin isoform in the liver, and *XG*, encoding a red blood cell surface protein (Xga). Two families with diabetes in which the men carried a 102-kb X deletion encompassing *GYG2* and part of *XG* have previously been described.[9] However, there was no clear co-segregation of the deletion with the diabetes phenotype, carrier males were in good physical health and reported no symptoms relating to fasting, and there was no significant change in their liver cell morphology. The only observed difference was a small rise in plasma glucose levels, suggesting that the phenotypic consequences of loss of *GYG2* are limited, perhaps as a result of compensating for the upregulation of the related autosomal gene *GYG1*, encoding the skeletal muscle isoform. This idea is supported by the observation that glycogen synthesis in GLN1-deficient individuals can be compensated by the production of GLN2.[34] The Xg blood group system does not appear to be of clinical relevance,[35] and approximately one-third of men and one-tenth of women of Northern European descent lack the Xga antigen, having the Xg-null phenotype, Xg(a−).[36] The vast majority of Xg(a−) individuals carry an X-linked SNP disrupting a GATA-binding motif upstream of *XG*, thus abolishing transcription specifically in erythroid tissues.[37] However, *XG*-*GYG2* deletions have been noted as a rare systemic cause of the Xg(a−) phenotype in both Swedish and Japanese Xg(a−) carriers.[38,39] Examination of the partial junction structures reported in Lee et al.[38] indicates that these deletions are a result of recurrent NAHR between the LTR6B elements implicated in this study and include at least one additional junction type to those described here (data not shown).

We were also able to carry out a PheWAS among the observed ePAR men, for whom the sample size is larger than the X-deletion individuals. Since *GYG2* is known to undergo X inactivation, at least in fibroblasts,[40,41] we reasoned that males carrying the ePAR are likely to have twice the female level of GLN2. No significant associations were noted with traits such as diabetes, liver function, or glycogen metabolism, as might have been expected.[9] Instead, even after correction for multiple testing, associations were noted including qualitative musculoskeletal phenotypes as well as a strong biochemical signal associated with circulating IGF1 levels, itself a robust determinant of skeletal health.[42] Further work is required to better understand the basis of these associations.

Aside from potential gene dosage effects, these rearrangements have the potential to impact sex chromosome pairing during meiosis. For men with the X deletion, the hybrid LTR6B element acts as the pseudoautosomal boundary—i.e., the limit of homology with the Y chromosome is effectively reduced by ~5 kb (19%) compared with normal men (see Figure 1). Although entire deletions of the PAR1 have been associated with male infertility,[43,44] it has been shown that deletion of the distal part of PAR1, leaving ~1.44 Mb of strict X-Y homology, still supports crossover as observed by the deletion swapping from the Y chromosome to an X chromosome within a family.[45] It therefore seems unlikely that the X deletions characterized here will have a noticeable effect on male fertility. Likewise, the ~115-kb X deletion is unlikely to have a significant effect on sex chromosome pairing in carrier females, as this deletion represents just 0.07% of the total length of the human X chromosome, still leaving almost 156 Mb of homology across which X-X crossover can be achieved.

We have already established via sperm-based analysis that the extended region of homology afforded by the ePAR supports crossover, and we estimated a minimum rate of crossover over the entire ePAR region of 6-fold greater than the genome average, comparable to pedigree estimates of PAR1 activity in general.[8] A recent study describing the *de novo* assembly of 43 Y chromosomes spanning 182,900 years of human evolution noted low diversity in the most proximal 500 kb of PAR1 and interpreted this to indicate low recombination in this region,[46] raising doubts about the true position of the pseudoautosomal boundary. If this were so, it would mean that the ePAR segment and its recombination hotspots might change the crossover behavior of the natural XY pair considerably. However, a re-examination of early evidence and analysis of PAR1 crossover in genomic datasets has confirmed the conventional position of the boundary.[47]

A number of other studies concerned with the sex chromosomes have recently made use of the UKB, for example, the general phenotypic effects of 47,XXY and 47,XYY karyotypes,[48] aneuploidy effects on venous thromboembolism,[49] and an association study of Y-Hgs with coronary artery disease.[13] However, these have focused on the White British ancestry cohort only, while we have gained insights by also examining the UK's minority ethnic groups, with their characteristically different Y-Hg spectra (Table S4). Ours also studied a Y chromosome rearrangement; in principle, other studies of Y deletions and duplications are possible, but any approach based on SNP microarray intensity would be limited by the physical distribution of the ~800 Y SNPs typed by the UKB. For example, characterization of the structural rearrangements that lead to deletion of *AMELY*,[50] a locus routinely used in DNA forensic testing to determine sex, would be amenable to analysis using UKB SNP-chip data. This is also the case for the *AZF*a and

-b regions, deletions of which are associated with male infertility.[51] In contrast, analysis of the most commonly deleted and dynamic region, *AZF*c, known to be important in male fertility, would not, as there are effectively no suitably located markers on the UKB Axiom array (Figure S7). However, with the release of whole-genome sequences for the UKB, such studies should now become possible.

Data and code availability

The published article includes all datasets generated during this study. The code generated during this study is available at https://github.com/EalesLabCompBio/extended-par-ukb.

Acknowledgments

This research was conducted using the UK Biobank Resource under application no. 32497 ("Numerical abnormalities and structural rearrangements of the Y and X chromosomes and predisposition to complex diseases") and supported by access to the high-performance computing facilities at the Universities of Leicester (ALICE) and Manchester (DPSF and CSF). We thank Veryan Codd and Nilesh Samani for assistance with access to UK Biobank DNA samples under an MTA from main application no. 6077 and Khaled Farah for laboratory work. The study was supported by British Heart Foundation grants PG/16/49/32176 and PG/12/9/29376 (to M.T.) and by a PhD grant to N.P. from the Faculty of Medicine Siriraj Hospital, Mahidol University, Thailand. For the purpose of open access, the author has applied a Creative Commons attribution license (CC BY) to any author-accepted manuscript version arising from this submission.

Author contributions

Conceptualization, M.A.J. and C.A.M.; formal analysis, N.P., J.E., D.S., C.A.M., X.X., and J.H.W.; investigation, N.P. and R.N.; writing – original draft, M.A.J., C.A.M., and J.H.W.; writing – review & editing, all authors; visualization, J.E., D.S., C.A.M., J.H.W., and M.A.J.; supervision, M.A.J. and C.A.M.; funding acquisition, M.T.

Declaration of interests

The authors declare no competing interests.

Supplemental information

Supplemental information can be found online at https://doi.org/10.1016/j.ajhg.2025.01.026.

Received: June 28, 2024
Accepted: January 31, 2025
Published: February 20, 2025

References

1. Cooke, H.J., Brown, W.R., and Rappold, G.A. (1985). Hypervariable telomeric sequences from the human sex chromosomes are pseudoautosomal. Nature *317*, 687–692. https://doi.org/10.1038/317687a0.
2. Simmler, M.C., Rouyer, F., Vergnaud, G., Nyström-Lahti, M., Ngo, K.Y., de la Chapelle, A., and Weissenbach, J. (1985). Pseudoautosomal DNA sequences in the pairing region of the human sex chromosomes. Nature *317*, 692–697. https://doi.org/10.1038/317692a0.
3. Burgoyne, P.S. (1982). Genetic homology and crossing over in the X and Y chromosomes of Mammals. Hum. Genet. *61*, 85–90. https://doi.org/10.1007/BF00274192.
4. Hassold, T.J., Sherman, S.L., Pettay, D., Page, D.C., and Jacobs, P.A. (1991). XY chromosome non-disjunction in man is associated with diminished recombination in the pseudoautosomal region. Am. J. Hum. Genet. *49*, 253–260.
5. Ellis, N., Yen, P., Neiswanger, K., Shapiro, L.J., and Goodfellow, P.N. (1990). Evolution of the pseudoautosomal boundary in Old World monkeys and great apes. Cell *63*, 977–986. https://doi.org/10.1016/0092-8674(90)90501-5.
6. Bachtrog, D. (2013). Y-chromosome evolution: emerging insights into processes of Y-chromosome degeneration. Nat. Rev. Genet. *14*, 113–124. https://doi.org/10.1038/nrg3366.
7. Mensah, M.A., Hestand, M.S., Larmuseau, M.H.D., Isrie, M., Vanderheyden, N., Declercq, M., Souche, E.L., Van Houdt, J., Stoeva, R., Van Esch, H., et al. (2014). Pseudoautosomal region 1 length polymorphism in the human population. PLoS Genet. *10*, e1004578. https://doi.org/10.1371/journal.pgen.1004578.
8. Poriswanish, N., Neumann, R., Wetton, J.H., Wagstaff, J., Larmuseau, M.H.D., Jobling, M.A., and May, C.A. (2018). Recombination hotspots in an extended human pseudoautosomal domain predicted from double-strand break maps and characterized by sperm-based crossover analysis. PLoS Genet. *14*, e1007680. https://doi.org/10.1371/journal.pgen.1007680.
9. Irgens, H.U., Fjeld, K., Johansson, B.B., Ringdal, M., Immervoll, H., Leh, S., Søvik, O., Johansson, S., Molven, A., and Njølstad, P.R. (2015). Glycogenin-2 is dispensable for liver glycogen synthesis and glucagon-stimulated glucose release. J. Clin. Endocrinol. Metab. *100*, E767–E775. https://doi.org/10.1210/jc.2014-4337.
10. Stankiewicz, P., and Lupski, J.R. (2006). The genomic basis of disease, mechanisms and assays for genomic disorders. Genome Dyn. *1*, 1–16. https://doi.org/10.1159/000092496.
11. Sudlow, C., Gallacher, J., Allen, N., Beral, V., Burton, P., Danesh, J., Downey, P., Elliott, P., Green, J., Landray, M., et al. (2015). UK biobank: an open access resource for identifying the causes of a wide range of complex diseases of middle and old age. PLoS Med. *12*, e1001779. https://doi.org/10.1371/journal.pmed.1001779.
12. Bycroft, C., Freeman, C., Petkova, D., Band, G., Elliott, L.T., Sharp, K., Motyer, A., Vukcevic, D., Delaneau, O., O'Connell, J., et al. (2018). The UK Biobank resource with deep phenotyping and genomic data. Nature *562*, 203–209. https://doi.org/10.1038/s41586-018-0579-z.
13. Eales, J.M., Maan, A.A., Xu, X., Michoel, T., Hallast, P., Batini, C., Zadik, D., Prestes, P.R., Molina, E., Denniff, M., et al. (2019). Human Y Chromosome Exerts Pleiotropic Effects on Susceptibility to Atherosclerosis. Arterioscler. Thromb. Vasc. Biol. *39*, 2386–2401. https://doi.org/10.1161/ATVBAHA.119.312405.
14. Collins, R.L., Brand, H., Karczewski, K.J., Zhao, X., Alföldi, J., Francioli, L.C., Khera, A.V., Lowther, C., Gauthier, L.D., Wang, H., et al. (2020). A structural variation reference for medical and population genetics. Nature *581*, 444–451. https://doi.org/10.1038/s41586-020-2287-8.

15. Athey, T.W. (2006). Haplogroup prediction from Y-STR values using a Bayesian-allele-frequency approach. J. Genet. Geneal. *2*, 34–39.
16. Bandelt, H.J., Forster, P., and Röhl, A. (1999). Median-joining networks for inferring intraspecific phylogenies. Mol. Biol. Evol. *16*, 37–48. https://doi.org/10.1093/oxfordjournals.molbev.a026036.
17. Lall, G.M., Larmuseau, M.H.D., Wetton, J.H., Batini, C., Hallast, P., Huszar, T.I., Zadik, D., Aase, S., Baker, T., Balaresque, P., et al. (2021). Subdividing Y-chromosome haplogroup R1a1 reveals Norse Viking dispersal lineages in Britain. Eur. J. Hum. Genet. *29*, 512–523. https://doi.org/10.1038/s41431-020-00747-z.
18. Wang, Q., Dhindsa, R.S., Carss, K., Harper, A.R., Nag, A., Tachmazidou, I., Vitsios, D., Deevi, S.V.V., Mackay, A., Muthas, D., et al. (2021). Rare variant contribution to human disease in 281,104 UK Biobank exomes. Nature *597*, 527–532. https://doi.org/10.1038/s41586-021-03855-y.
19. Poznik, G.D., Xue, Y., Mendez, F.L., Willems, T.F., Massaia, A., Wilson Sayres, M.A., Ayub, Q., McCarthy, S.A., Narechania, A., Kashin, S., et al. (2016). Punctuated bursts in human male demography inferred from 1,244 worldwide Y-chromosome sequences. Nat. Genet. *48*, 593–599. https://doi.org/10.1038/ng.3559.
20. Graffelman, J., and Weir, B.S. (2016). Testing for Hardy-Weinberg equilibrium at biallelic genetic markers on the X chromosome. Heredity *116*, 558–568. https://doi.org/10.1038/hdy.2016.20.
21. Poriswanish, N. (2018). PhD thesis: Recombination Behaviour and Evolutionary History of the Extended Pseudoautosomal Region 1 (ePAR) on the Human Y chromosome; https://lra.le.ac.uk/handle/2381/43219. PhD (University of Leicester).
22. 1000 Genomes Project Consortium, Auton, A., Brooks, L.D., Durbin, R.M., Garrison, E.P., Kang, H.M., Korbel, J.O., Marchini, J.L., McCarthy, S., McVean, G.A., and Abecasis, G.R. (2015). A global reference for human genetic variation. Nature *526*, 68–74. https://doi.org/10.1038/nature15393.
23. Repping, S., van Daalen, S.K.M., Brown, L.G., Korver, C.M., Lange, J., Marszalek, J.D., Pyntikova, T., van der Veen, F., Skaletsky, H., Page, D.C., and Rozen, S. (2006). High mutation rates have driven extensive structural polymorphism among human Y chromosomes. Nat. Genet. *38*, 463–467. https://doi.org/10.1038/ng1754.
24. Balaresque, P., Bowden, G.R., Parkin, E.J., Omran, G.A., Heyer, E., Quintana-Murci, L., Roewer, L., Stoneking, M., Nasidze, I., Carvalho-Silva, D.R., et al. (2008). Dynamic nature of the proximal AZFc region of the human Y chromosome: multiple independent deletion and duplication events revealed by microsatellite analysis. Hum. Mutat. *29*, 1171–1180. https://doi.org/10.1002/humu.20757.
25. Fenner, J.N. (2005). Cross-cultural estimation of the human generation interval for use in genetics-based population divergence studies. Am. J. Phys. Anthropol. *128*, 415–423. https://doi.org/10.1002/ajpa.20188.
26. Turner, D.J., Miretti, M., Rajan, D., Fiegler, H., Carter, N.P., Blayney, M.L., Beck, S., and Hurles, M.E. (2008). Germline rates of de novo meiotic deletions and duplications causing several genomic disorders. Nat. Genet. *40*, 90–95. https://doi.org/10.1038/ng.2007.40.
27. Batini, C., and Jobling, M.A. (2017). Detecting past male-mediated expansions using the Y chromosome. Hum. Genet. *136*, 547–557. https://doi.org/10.1007/s00439-017-1781-z.
28. Millett, M. (1990). The Romanization of Britain : An Essay in Archaeological Interpretation (Cambridge University Press).
29. Charchar, F.J., Bloomer, L.D., Barnes, T.A., Cowley, M.J., Nelson, C.P., Wang, Y., Denniff, M., Debiec, R., Christofidou, P., Nankervis, S., et al. (2012). Inheritance of coronary artery disease in men: an analysis of the role of the Y chromosome. Lancet *379*, 915–922. https://doi.org/10.1016/S0140-6736(11)61453-0.
30. Maan, A.A., Eales, J., Akbarov, A., Rowland, J., Xu, X., Jobling, M.A., Charchar, F.J., and Tomaszewski, M. (2017). The Y chromosome: a blueprint for men's health? Eur. J. Hum. Genet. *25*, 1181–1188. https://doi.org/10.1038/ejhg.2017.128.
31. Scholz, M., Horn, K., Pott, J., Wuttke, M., Kühnapfel, A., Nasr, M.K., Kirsten, H., Li, Y., Hoppmann, A., Gorski, M., et al. (2024). X-chromosome and kidney function: evidence from a multi-trait genetic analysis of 908,697 individuals reveals sex-specific and sex-differential findings in genes regulated by androgen response elements. Nat. Commun. *15*, 586. https://doi.org/10.1038/s41467-024-44709-1.
32. Millard, L.A.C., Davies, N.M., Gaunt, T.R., Davey Smith, G., and Tilling, K. (2018). Software Application Profile: PHESANT: a tool for performing automated phenome scans in UK Biobank. Int. J. Epidemiol. *47*, 29–35. https://doi.org/10.1093/ije/dyx204.
33. Zhou, Y., Zhan, X., Jin, J., Zhou, L., Bergman, J., Li, X., Rousselle, M.M.C., Belles, M.R., Zhao, L., Fang, M., et al. (2023). Eighty million years of rapid evolution of the primate Y chromosome. Nat. Ecol. Evol. *7*, 1114–1130. https://doi.org/10.1038/s41559-022-01974-x.
34. Krag, T.O., Ruiz-Ruiz, C., and Vissing, J. (2017). Glycogen Synthesis in Glycogenin 1-Deficient Patients: A Role for Glycogenin 2 in Muscle. J. Clin. Endocrinol. Metab. *102*, 2690–2700. https://doi.org/10.1210/jc.2017-00399.
35. Johnson, N.C. (2011). XG: the forgotten blood group system. Immunohematol *27*, 68–71.
36. Daniels, G. (2013). Human Blood Groups, 3rd Edition (John Wiley & Sons, Ltd.).
37. Möller, M., Lee, Y.Q., Vidovic, K., Kjellström, S., Björkman, L., Storry, J.R., and Olsson, M.L. (2018). Disruption of a GATA1-binding motif upstream of XG/PBDX abolishes Xg(a) expression and resolves the Xg blood group system. Blood *132*, 334–338. https://doi.org/10.1182/blood-2018-03-842542.
38. Lee, Y.Q., Storry, J.R., Karamatic Crew, V., Halverson, G.R., Thornton, N., and Olsson, M.L. (2019). A large deletion spanning XG and GYG2 constitutes a genetic basis of the Xg(null) phenotype, underlying anti-Xg(a) production. Transfusion *59*, 1843–1849. https://doi.org/10.1111/trf.15242.
39. Watanabe-Okochi, N., Uchikawa, M., Tsuneyama, H., Ogasawara, K., Shiraishi, R., Masuno, A., Onodera, T., Tsuno, N.H., and Muroi, K. (2022). Genetic background of anti-Xg(a) producers in Japanese blood donors. Vox Sang. *117*, 1235–1239. https://doi.org/10.1111/vox.13342.
40. Carrel, L., and Willard, H.F. (2005). X-inactivation profile reveals extensive variability in X-linked gene expression in females. Nature *434*, 400–404. https://doi.org/10.1038/nature03479.
41. Stabellini, R., Vasques, L.R., de Mello, J.C.M., Hernandes, L.M., and Pereira, L.V. (2009). MAOA and GYG2 are submitted to X chromosome inactivation in human fibroblasts. Epigenetics *4*, 388–393. https://doi.org/10.4161/epi.4.6.9492.
42. Mazziotti, G., Lania, A.G., and Canalis, E. (2022). Skeletal disorders associated with the growth hormone-insulin-like

growth factor 1 axis. Nat. Rev. Endocrinol. *18*, 353–365. https://doi.org/10.1038/s41574-022-00649-8.

43. Gabriel-Robez, O., Rumpler, Y., Ratomponirina, C., Petit, C., Levilliers, J., Croquette, M.F., and Couturier, J. (1990). Deletion of the pseudoautosomal region and lack of sex-chromosome pairing at pachytene in two infertile men carrying an X;Y translocation. Cytogenet. Cell Genet. *54*, 38–42. https://doi.org/10.1159/000132951.
44. Mohandas, T.K., Speed, R.M., Passage, M.B., Yen, P.H., Chandley, A.C., and Shapiro, L.J. (1992). Role of the pseudoautosomal region in sex-chromosome pairing during male meiosis: meiotic studies in a man with a deletion of distal Xp. Am. J. Hum. Genet. *51*, 526–533.
45. Fukami, M., Fujisawa, Y., Ono, H., Jinno, T., and Ogata, T. (2020). Human Spermatogenesis Tolerates Massive Size Reduction of the Pseudoautosomal Region. Genome Biol. Evol. *12*, 1961–1964. https://doi.org/10.1093/gbe/evaa168.
46. Hallast, P., Ebert, P., Loftus, M., Yilmaz, F., Audano, P.A., Logsdon, G.A., Bonder, M.J., Zhou, W., Höps, W., Kim, K., et al. (2023). Assembly of 43 human Y chromosomes reveals extensive complexity and variation. Nature *621*, 355–364. https://doi.org/10.1038/s41586-023-06425-6.
47. Bellott, D.W., Hughes, J.F., Skaletsky, H., Owen, E.C., and Page, D.C. (2024). Where is the boundary of the human pseudoautosomal region? Am. J. Hum. Genet. *111*, 2530–2541. https://doi.org/10.1016/j.ajhg.2024.09.005.
48. Zhao, Y., Gardner, E.J., Tuke, M.A., Zhang, H., Pietzner, M., Koprulu, M., Jia, R.Y., Ruth, K.S., Wood, A.R., Beaumont, R.N., et al. (2022). Detection and characterization of male sex chromosome abnormalities in the UK Biobank study. Genet. Med. *24*, 1909–1919. https://doi.org/10.1016/j.gim.2022.05.011.
49. Berry, A.S.F., Finucane, B.M., Myers, S.M., Abril, A., Kirchner, H.L., Ledbetter, D.H., Martin, C.L., and Oetjens, M.T. (2023). Association of Supernumerary Sex Chromosome Aneuploidies With Venous Thromboembolism. JAMA *329*, 235–243. https://doi.org/10.1001/jama.2022.23897.
50. Jobling, M.A., Lo, I.C.C., Turner, D.J., Bowden, G.R., Lee, A.C., Xue, Y., Carvalho-Silva, D., Hurles, M.E., Adams, S.M., Chang, Y.M., et al. (2007). Structural variation on the short arm of the human Y chromosome: recurrent multigene deletions encompassing *Amelogenin Y*. Hum. Mol. Genet. *16*, 307–316. https://doi.org/10.1093/hmg/ddl465.
51. Vogt, P.H. (2005). AZF deletions and Y chromosomal haplogroups: history and update based on sequence. Hum. Reprod. Update *11*, 319–336. https://doi.org/10.1093/humupd/dmi017.

The American Journal of Human Genetics, Volume *112*

Supplemental information

Multiple origins and phenotypic implications of an extended human pseudoautosomal region shown by analysis of the UK Biobank

Nitikorn Poriswanish, James Eales, Xiaoguang Xu, David Scannali, Rita Neumann, Jon H. Wetton, Maciej Tomaszewski, Mark A. Jobling, and Celia A. May

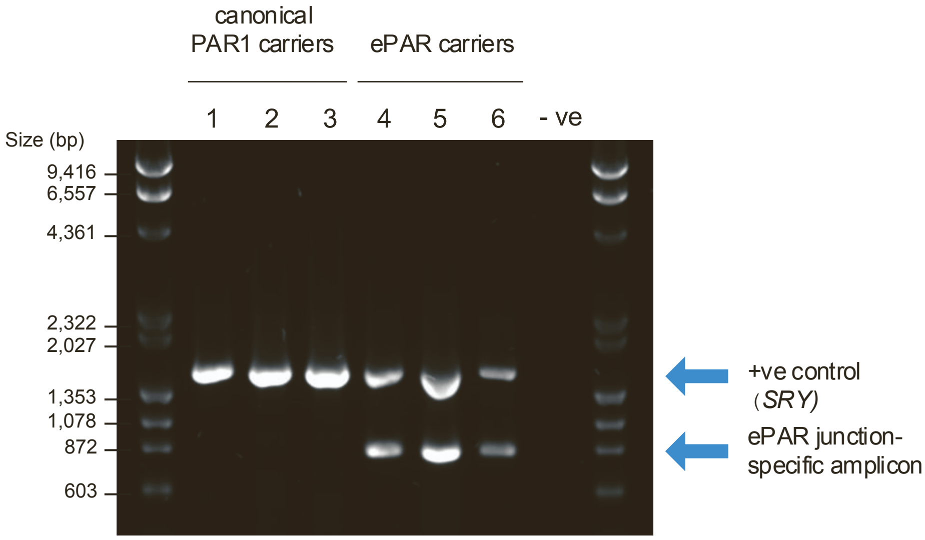

Figure S1: PCR assay for the detection of ePAR-bearing Y chromosomes.

Ethidium-stained agarose gel showing PCR products generated in three normal (canonical PAR1) males and three ePAR-carrying males. The smaller PCR product derives from the ePAR junction sequence, and the larger from the Y-specific *SRY* gene, acting as control for successful PCR.

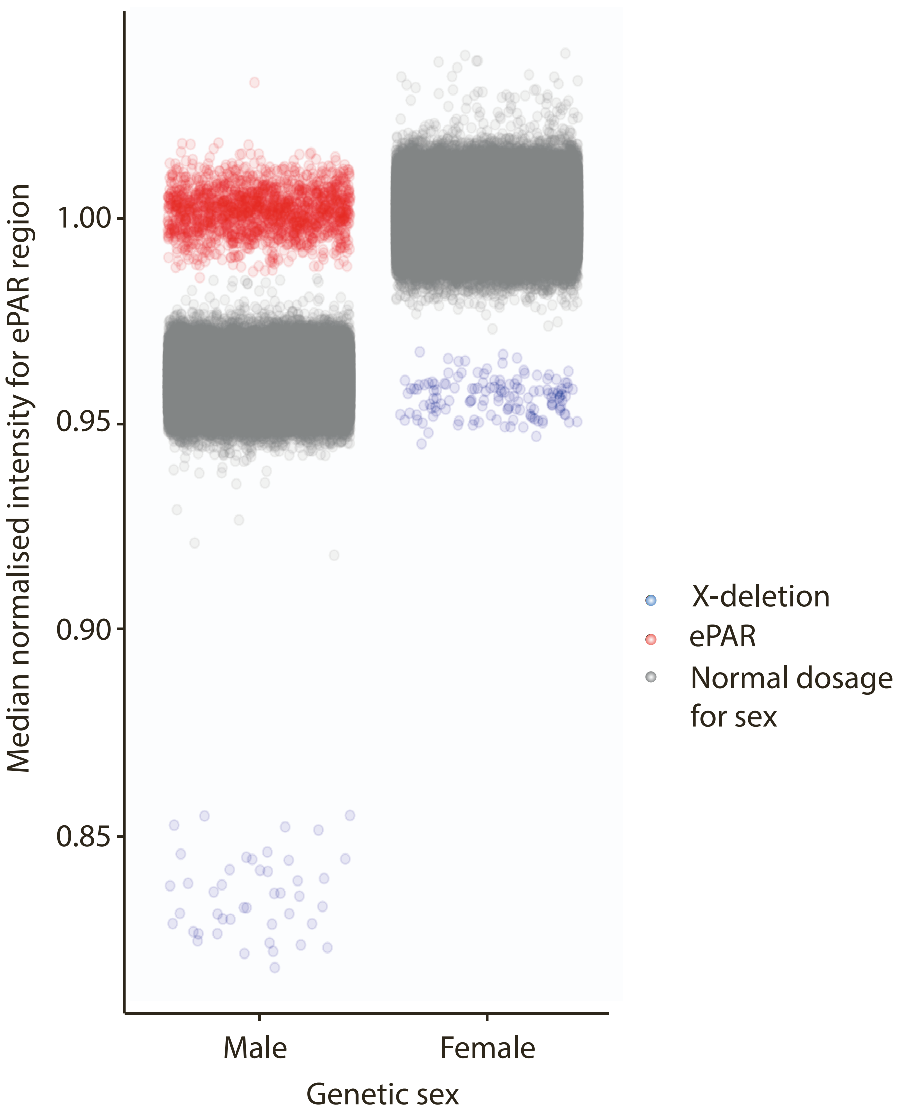

Figure S2: Median normalised intensity data for ePAR-informative SNPs amongst UK Biobank 46,XY males and 46,XX females.

The cloud of blue points in females signifies likely heterozygous carriers of the X-deletion (n=137). No female homozygotes are evident. Compare to Figure 2, in which males are subdivided by Y-hg.

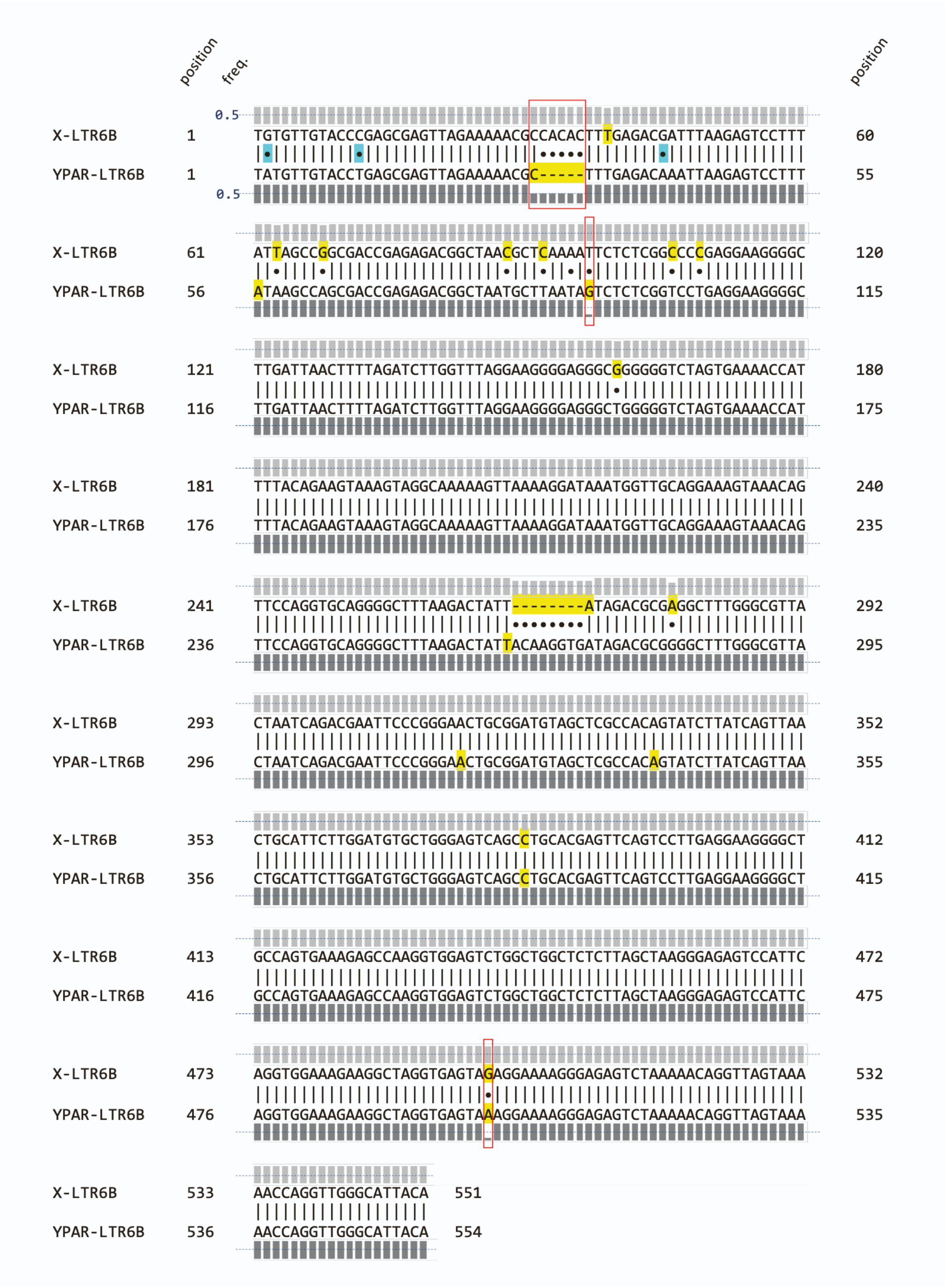

Figure S3: Alignment of the LTR6B elements from the X chromosome and Y-PAR that are implicated in the generation of the ePAR.

Alignments are shown between the hg38 genome references chrX:2,890,506-2,891,056 and chrY:2,776,108-2,776,661 with the associated histograms indicating the frequency of each base. Mismatches highlighted in cyan correspond to fixed differences between the two elements and nucleotides highlighted in yellow correspond to sites of known variation observed from the 1000 Genomes Project. The red boxes highlight instances where a reference sequence is most likely to lead to misinterpretation of NAHR-mediated junctions within ePAR candidates and the reciprocal deletion events.

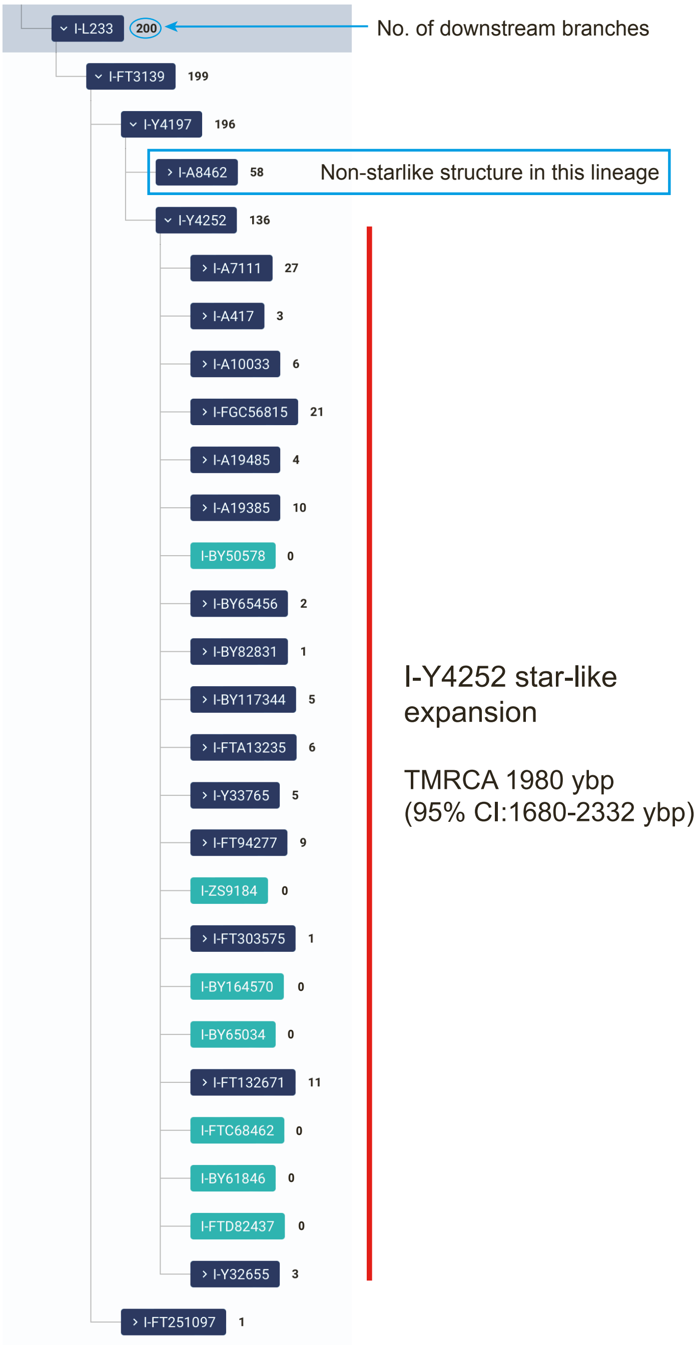

Figure S4: High-resolution SNP phylogeny for the major ePAR-bearing lineage I2-L233.

Part of the FamilyTreeDNA Y Haplotree showing the Y-Hg I2-L233 phylogeny based on 'Big Y' direct-to-consumer testing, which sequences 16-22.5 Mb of Y-chromosomal DNA for SNP discovery. Sublineages are named by their terminal SNPs. A striking star-like expansion, containing at least 26 distinct sub-lineages (the 22 shown, plus four as yet unnamed sub-lineages) descends from the I-Y4252 node in the Haplotree. Dates for the TMRCA of this expansion (estimated by FamilyTreeDNA using a relaxed clock model) match closely those estimated independently in this study for Y-Hg I2a_L233 from PPY23 Y-STR haplotypes. The precise position of the ePAR occurrence in the phylogeny is unclear, but we have observed ePAR carriers among those derived for SNP Y4252 and A8462. This lineage shares a common ancestor, Y4197, with a TMRCA of 150 BCE, which accounts for 98% of typed I-L233 individuals (data not shown). The contributing DNA donors have NW European / British Isles ancestry where this is stated.

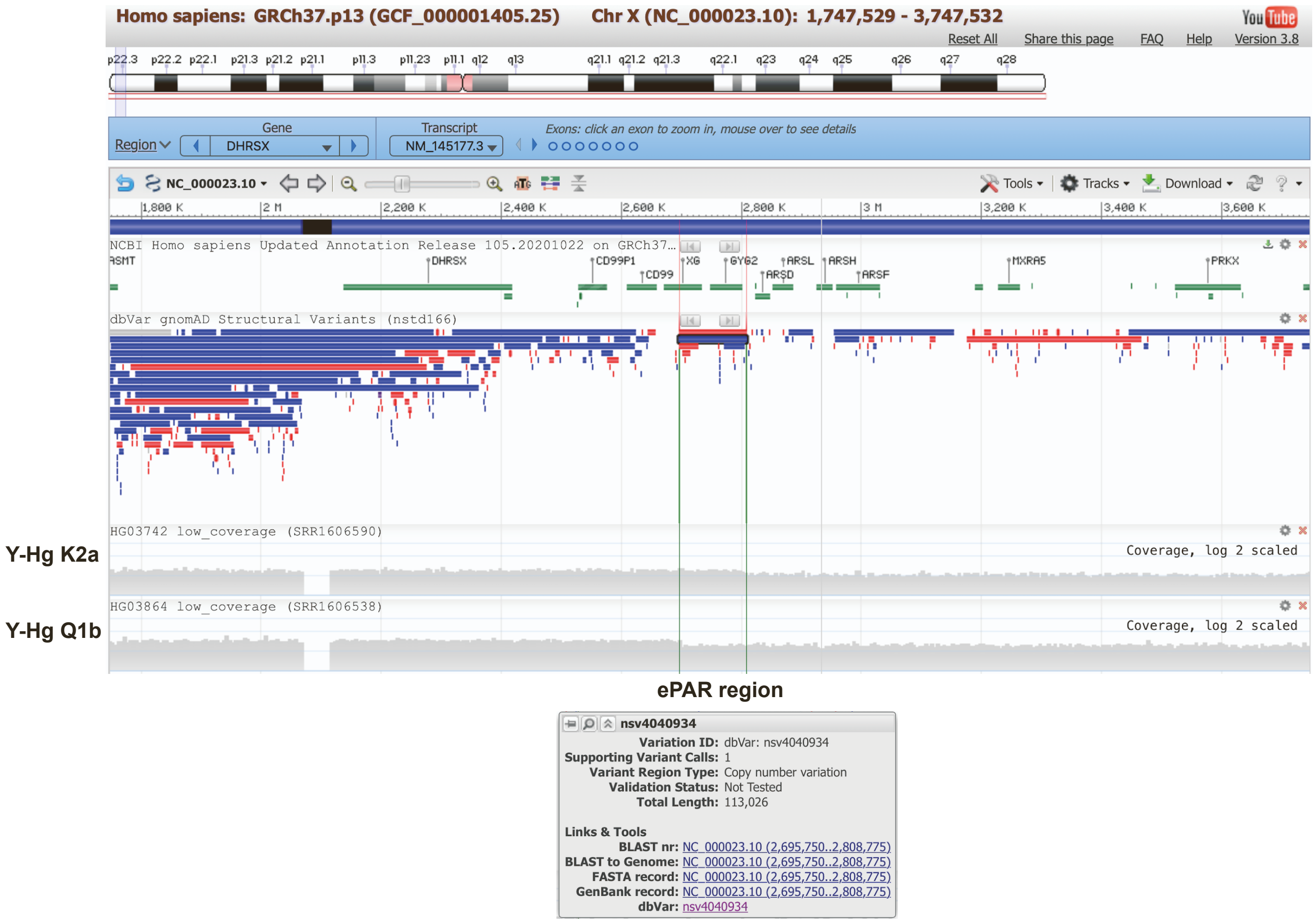

Figure S5: ePAR in a Y-Hg K2a 1000 Genomes Project male detected via sequence coverage

A screengrab of the 1000 Genomes Project browser (ncbi.nlm.nih.gov/variation/tools/1000genomes/ - now retired, but accessed 25/06/2021) centred on the interval corresponding to ePAR. HG03742 (Y-Hg K2a) shows elevated sequence coverage in this interval compared to a control (HG03864; Y-Hg Q1b). Haplogroup information is from Poznik et al. (2016).

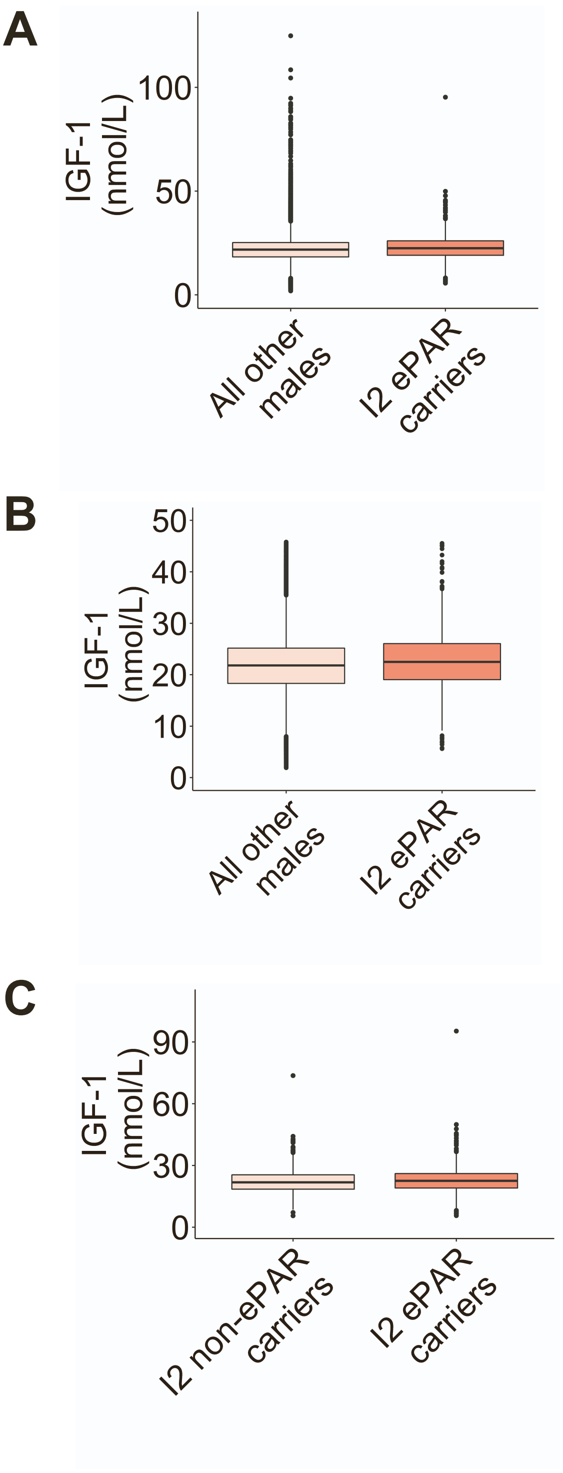

Figure S6: Levels of IGF-1 in I2 ePAR carriers compared to non-ePAR carriers.

A: Circulating IGF-1 levels in I2 ePAR carriers compared with all other non-ePAR men in the UKBiobank cohort. **B**: As (A), but excluding outliers. **C**: Persistence of the effect when comparisons are restricted among White British men of Y-Hg I2. Statistics accompanying these comparisons are given in Table S8.

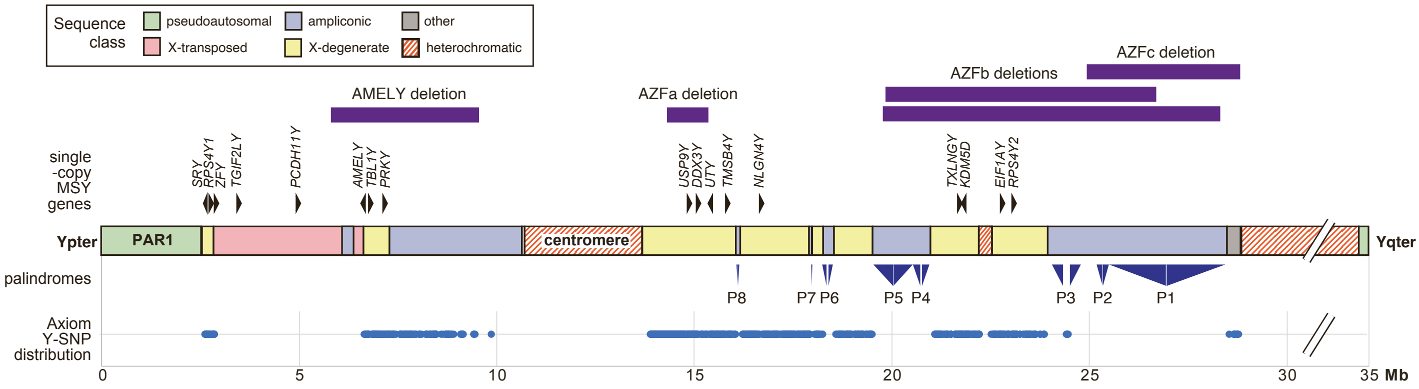

Figure S7: Distribution on MSY of Y-SNPs on the UK Biobank Axiom array.

Positions of the 813 Y-SNPs are shown relative to a schematic MSY structure based on that of Skaletsky et al. (2003). Also shown are the positions of single-copy genes, and known large deletions. The AZFc deletion will not be detectable based on SNP array data due to a lack of SNPs in the deleted region.
